# Advances in the evolution of antibiotic resistance risks in hospital wastewater and multibarrier control strategies

**DOI:** 10.3389/fmicb.2026.1789579

**Published:** 2026-04-20

**Authors:** Zhankun Zhu, Jinfeng Shu, Yaoqin Zhang, Ying Gao, Kehong Lou, Guosheng Gao

**Affiliations:** 1 Department of General Affairs, Ningbo No. 2 Hospital, Ningbo, China; 2 Department of Clinical Laboratory, Ningbo No. 2 Hospital, Ningbo, China; 3 Zhejiang Renxin Testing Research Institute Co., Ltd., Ningbo, China

**Keywords:** antibiotic resistance, life-cycle risk governance, multi-barrier control, resistance genes (ARGs), risk evolution

## Abstract

Since it is imbued with antibiotics, resistant bacteria, and their resistance genes, hospital waste has transformed post-medical “tail water” into a global epicenter of connected ecological and health emergencies. By considering a “gap identification, risk tracking, and barrier rebuilding” framework and integrating 10 years of worldwide evidence, we first reveal how four mutually reinforcing deficits: absent primary treatment units, static design, aging infrastructure, and a hollowed-out workforce—perpetually overload small-scale facilities, unleashing high loads of antibiotics and antibiotics resistance genes (ARGs). We then follow ng/L residues along the “outfall—sediment—zooplankton—fish” continuum, showing how horizontal gene transfer (HGT) and mutational evolution processes restructure microbial communities, suppress algal photosynthesis and fish reproduction, and ultimately amplify threats to biodiversity and human health throughout the food web. To address the paradox that treatment does not equate to safety, we advance a multibarrier portfolio: (i) implement proactive retrofitting of equipment to confer inherent operational flexibility; (ii) process-stage adsorption-biodegradation hybrids that curtail selective pressure; and (iii) a harmonized, end-line monitoring network coupled with bioindicators to pinpoint ARG hotspots. Complementary measures, including regional pooled maintenance, microcredential training, green finance incentives, and a global data-sharing platform, shift the governance paradigm from end-of-pipe removal to life-cycle risk management, offering a replicable, technoinstitutional roadmap to overcome the pollution-resistance feedback loop.

## 1 Introduction

Hospitals, which play a vital role in safeguarding human health, produce complex and high-risk wastewater during the provision of diagnostic, therapeutic, and nursing services (Zhu et al., 2024). This wastewater contains not only high concentrations of pathogenic microorganisms and incompletely metabolized active pharmaceutical ingredients but also high-risk antibiotic-resistant bacteria, such as carbapenem-resistant *Klebsiella pneumoniae* (CRKP) and methicillin-resistant *Staphylococcus aureus* (MRSA), as well as antibiotic resistance genes (ARGs) (Wang C. et al., 2022). These elements pose a dual threat to public health and the ecological environment. As global medical demand continues to increase because of the aging population, changes in disease spectra, and the need to respond to public health events, the volume of hospital wastewater discharged is increasing annually. In particular, small institutions, which account for more than 70% of the total number of global healthcare facilities. Their weak infrastructure and insufficient operation and maintenance (O&M) capabilities further exacerbate environmental exposure risks (Zhang et al., 2020).

Despite notable advancements in the optimization of hospital wastewater treatment, pollutant detection, and risk assessment strategies on a global scale, substantial challenges persist in infrastructure configuration, core technology application, and management and maintenance practices (Gu L. et al., 2024). These challenges hinder the effective mitigation of critical issues, such as the dissemination of antibiotic-resistant bacteria and the accumulation of ARGs (Kang et al., 2024). In this context, a systematic review of the existing contradictions within hospital wastewater treatment systems is imperative. This review is focused on analyzing the environmental fates and health hazards of residual pollutants and elucidating the mutation mechanisms and dissemination pathways of ARGs. Such efforts are essential for overcoming existing governance challenges and facilitating a transformative shift in medical wastewater treatment from “passive compliance” to “proactive prevention and control” (Jiang Q. et al., 2023). In this context, we synthesize the latest global research findings from 2020 to 2025. Adhering to the logical framework of “facility deficiencies—technological bottlenecks—environmental risks—health threats,” this review provides an in-depth analysis of the core issues affecting hospital wastewater pollution control (Boutros et al., 2025). We aim to offer robust theoretical support and practical references for the development of targeted governance strategies and the enhancement of management systems. Ultimately, this work contributes to the full-chain, refined, and sustainable governance of hospital wastewater.

**FIGURE 1:**
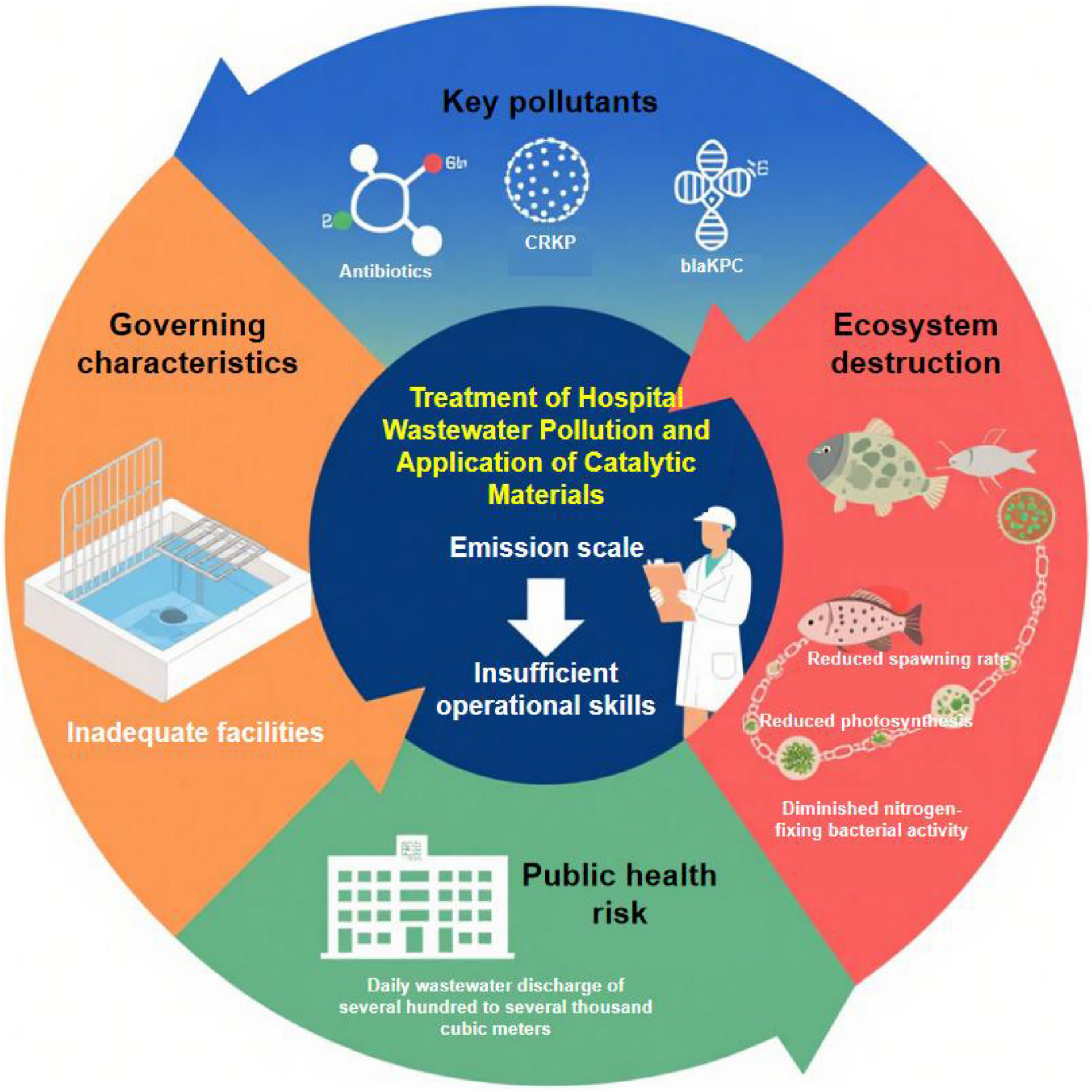
Hospital wastewater pollution: key pollutants, impacts, and catalytic materials in treatments.

Figure 1 presents hospital wastewater pollution control strategies and catalytic material applications, systematically presenting the full-chain logical framework of hospital wastewater pollution: core pollutants (antibiotics and the *bla*_*KPC*_ resistance gene) are discharged at a typical daily scale of hundreds to thousands of cubic meters, leading to ecosystem impairment (e.g., reduced fish spawning rates and diminished nitrogen-fixing bacterial activity) and public health risks. On the governance side, bottlenecks such as inadequate facility configuration and insufficient operational skills collectively form the core challenges and analytical framework for hospital wastewater pollution control.

## 2 Methods

### 2.1 Literature search strategy

This review conducted systematic searches in English databases including Web of Science, Scopus, and PubMed, using “hospital wastewater,” “antibiotic resistance,” “antibiotic resistance genes (ARGs),” “risk evolution,” and “control strategy” as core search terms. The search time range was from January 2016 to June 2025. Additionally, manual searches were performed for highly cited papers and reviews in relevant fields, as well as reference lists of cited literature, to ensure comprehensive literature retrieval.

### 2.2 Inclusion criteria

#### 2.2.1 Study types

Published English-language original research (including experimental studies, field investigations, cohort studies, and case analyses), systematic reviews, and meta-analyses.

#### 2.2.2 Study subjects

Studies focusing on antibiotic, antibiotic-resistant bacteria (ARB), and ARGs contamination in hospital wastewater, covering directions such as treatment system deficiencies, environmental fate, ecological and health risks, and control technologies.

#### 2.2.3 Study content

Research providing verifiable results, including original data related to hospital wastewater, risk evolution mechanisms, and effectiveness of control strategies.

#### 2.2.4 Publication requirements

Research outcomes published in SCI/SSCI-indexed journals with complete data and clear conclusions.

### 2.3 Exclusion criteria

Studies focusing on non-hospital wastewater, including but not limited to municipal sewage, agricultural and aquaculture wastewater, pharmaceutical manufacturing wastewater, and other single-source industrial effluents.

Studies involving antibiotic detection without addressing resistance risks, or investigating control technologies without considering the specific characteristics of hospital wastewater.

Conference abstracts, theses/dissertations, and unpublished gray literature characterized by incomplete datasets or ambiguous conclusions.

Duplicate publications and studies demonstrating low thematic relevance to the scope of this review.

### 2.4 Literature selection procedure

**FIGURE 2:**
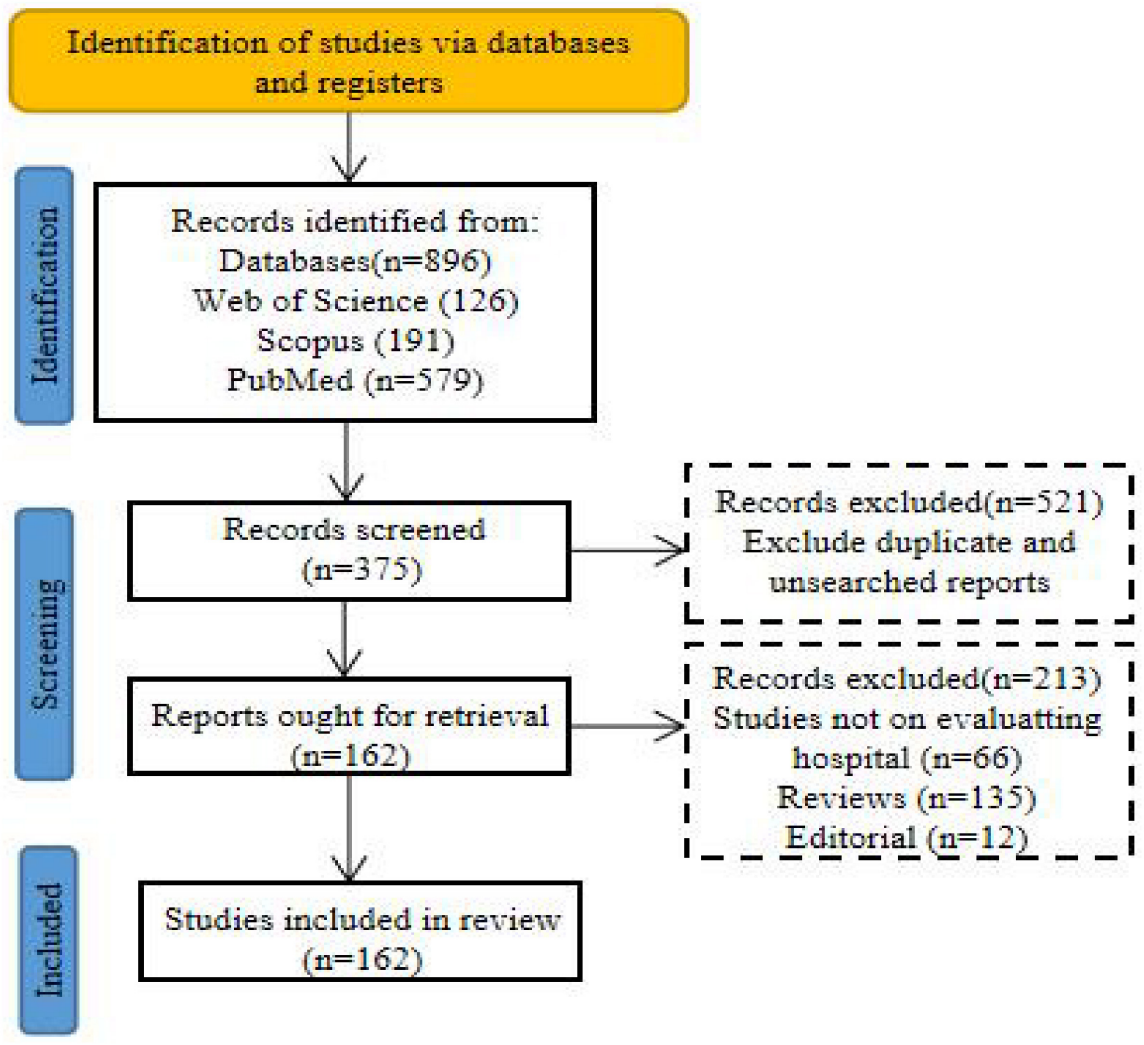
PRISMA study flowchart.

The literature screening process in this study strictly followed the PRISMA (Preferred Reporting Items for Systematic Reviews and Meta-Analyses) statement and was implemented in three phases: Phase 1: Initial screening: Two independent reviewers screened titles and abstracts to identify potentially relevant studies and exclude obviously ineligible records, with reasons for exclusion documented; Phase 2: Full-text eligibility assessment: Articles passing initial screening were retrieved for full-text review to determine final eligibility, with any disagreements between reviewers resolved through arbitration by a third senior reviewer to achieve consensus; Phase 3: Final selection and data extraction: Studies meeting all inclusion criteria underwent standardized data extraction, capturing information on study region, research subjects, experimental methodologies, primary results, and conclusions. Articles were excluded at this stage if they presented duplicate data or contained insufficient information for valid data extraction. A total of 896 records were identified through the initial search. After removing 521 records during the title and abstract screening and 213 articles during the full-text screening, 162 eligible studies were ultimately included, providing core evidence support for the analysis and conclusions of this review (Figure 2).

### 2.5 Evidence synthesis and analytical approach

#### 2.5.1 Evidence hierarchy

Included studies were stratified into three evidence tiers according to research design: Tier I—large-scale field investigations, randomized controlled experiments, and systematic reviews/meta-analyses; Tier II—small-scale field experiments, case studies, and cohort studies; Tier III—laboratory simulation studies and preliminary exploratory research. Priority was given to Tier I and Tier II evidence, while Tier III evidence was utilized as complementary reference.

#### 2.5.2 Thematic synthesis

Employing an analytical framework of “Gap Identification, Risk Tracking, and Barrier Reconstruction,” the included literature was thematically categorized to systematically synthesize research findings on treatment system deficiencies, environmental fate of resistant contaminants, ecological and health risks, and control strategies in hospital wastewater. Cross-validation of results across studies was performed; for contentious conclusions, explanatory analysis was conducted by integrating contextual factors including geographical study settings and methodological approaches.

#### 2.5.3 Quantitative data integration

Key parameters extracted from eligible studies, including antibiotic concentrations, ARGs abundance, and treatment technology removal efficiencies, were quantitatively synthesized. Comparative analyses were conducted to elucidate both universal patterns and regional variations in antimicrobial resistance risks associated with hospital wastewater globally, thereby furnishing empirical foundations for the development of multi-barrier control strategies.

## 3 High-risk inventory in hospital wastewater

To date, hospital waste is recognized as a trifecta of chemical, biological and radiological hazards (Table 1). The chemical spectrum is vast, including legacy heavy metals, inorganic toxins and a new wave of persistent organics, which are routinely detected. PFOA at the mg/L^–1^ scale perturbs lipid metabolism in fish, resulting in spinal curvature, pericardial edema and stunted growth. Antibiotics are the most biologically active components in waste: sulfamethoxazole, trimethoprim, ciprofloxacin, azithromycin, clarithromycin, metronidazole, vancomycin and ceftriaxone occur at the μg/L^–1^ scale in raw sewage, with peaks occurring at levels 3–5-fold higher in winter than in summer, suppressing nitrifiers and accelerating ARG enrichment under constant selective pressure conditions. Total coliforms are on the scale of 10^7^–10^10^ CFU/L^–1^, and *Salmonella*, *Shigella*, *Pseudomonas aeruginosa*, *Mycobacterium tuberculosis*, *noroviruses*, *rotaviruses* and *protozoan ova* undergo recurrent isolation. ARGs (*sul1*, *sul2*, *tetM*, *bla*_*TEM*_, *ermB*, and *qnrS*) are 1–2 orders of magnitude more abundant than in municipal wastewater, and they coexist with mobile elements (*intI1* and *ISCR2*), leading to the formation of a formidable “molecular library” for HGT. Radiologically, patient-excreted isotopes (^131^I, ^99m^Tc, ^18^F, and ^90^Y) enter sewers at ^131^I activity levels of 15–62 Bq/L^–1^. The environmental half-life of this type of substance can range from several days to several weeks, resulting in the formation of long-term pollution hotspots downstream., and increase the thyroid levels of riverside residents. Confronted by this chemical—biological—radiological triad, most hospital wastewater systems still mimic municipal plant designs, which feature ill-matched processes, lax operating set-points and absent regulatory metrics and thus legally breach final barriers.

**TABLE 1:** Hazardous substances in hospital wastewater.

Hazard dimension	Specific hazardous substances	Concentration level	Potential hazards	References
Chemical	Heavy metals and inorganic toxins: Hg, Cd, Cr(VI), Pb, As, Ni, cyanide, fluoride, nitrite	Specific concentration not specified; typical hazardous substances in traditional cognition.	Core category of chemical hazards in hospital wastewater.	Pasquini et al., 2014; Chen et al., 2017; Alam et al., 2022
	Persistent organic pollutants: nonylphenol, perfluorooctanoic acid (PFOA), perfluorooctane sulfonate (PFOS), phthalates, polybrominated diphenyl ethers, etc. (related to COD).	PFOA concentration reaches mg⋅L^–1^ level.	PFOA can interfere with lipid metabolism in fish and induce typical malformations in animals.	Sun et al., 2023
	Antibiotics: sulfamethoxazole, trimethoprim, ciprofloxacin, azithromycin, etc.	Concentration in raw wastewater is generally at μg⋅L^–1^ level	Inhibits the activity of nitrifying bacteria in water bodies and accelerates the enrichment of antibiotic resistance genes through selective pressure.	Al Qarni et al., 2016; Lamba et al., 2017; Gönder et al., 2021; Silvester et al., 2025)
Biological	Pathogenic microorganisms: total coliforms, salmonella, shigella, etc.	1. Total coliforms: 10^7^–10^10^ CFU⋅L^–1^; 2. Abundance of antibiotic resistance genes is 1–2 orders of magnitude higher than that in urban sewage.	Horizontal gene transfer, spreading pathogenic microorganisms and antibiotic resistance genes.	(Anssour et al., 2016; Pires et al., 2023
Radiological	Radioactive isotopes: ^131^I, ^99m^Tc, ^18^F, ^90^Y, etc. (mainly from nuclear medicine department).	^131^I activity ranges from 15–62 Bq⋅L^–1^ with a half-life of several days.	Increasing the internal thyroid irradiation dose of residents along the coast.	Rodríguez, 2012; Martinez et al., 2014; Liu J. et al., 2025

## 4 Current issues in hospital wastewater treatment systems

Over the past 5 years, global investment in health infrastructure has predominantly concentrated on large urban wastewater treatment plants and high-end medical complexes, but the substantial number of small medical institutions with fewer than 200 beds has been overlooked. These data indicate that these small institutions account for 28% of global medical wastewater but only 6% of wastewater treatment investment. This structural imbalance has continuously exacerbated the quadruple dilemma of “facility absence, capacity mismatch, equipment aging, and human resource shortage.”

### 4.1 Incomplete wastewater treatment facilities

The 2024 Global Review identified the absence of primary treatment units as the top risk; this characteristic originated from structural biases in decision-making (Puga, 2024). Although screens, grit chambers, and equalization tanks are fundamental processes, they are often the first to be abandoned during budget cuts due to their inability to directly reduce chemical oxygen demand (COD) and their difficulty in attracting external financing. Consequently, a “visibility paradox” is created: foundational and hard-to-quantify processes are easily overlooked, while flashy and visible technologies are more likely to receive investment. Once operations commence, the absence of screens leads to cotton swabs and plastic fragments entangling aeration discs, the lack of an equalization tank causes pH fluctuations to impact biochemical units, and the absence of a grit chamber results in sand abrasion on pump bodies. At this point, the remediation cost is 3–5 times the initial investment, with additional operational instability. Moreover, managers often inaccurately consider the absence of catastrophic events as a controllable risk, thereby delaying remediation efforts until a disaster occurs. Only by incorporating indicators related to primary treatment units into the life-cycle cost model and quantifying their potential health expenditures can these “silent units” affect decision-making processes.

Experimental data indicate that the fecal coliform concentration in the effluent of broken-link facilities can reach 7.2 log CFU/100 mL (Bhandari et al., 2023). This alarming level is attributed to a systemic collapse of pathogen barriers, which follows a three-step process of “broken link, imbalance, and loss of control.” The absence of screens allows debris to enter the biochemical tank, where it entangles aeration equipment and creates local anaerobic conditions. The lack of an equalization tank causes the pH value to fluctuate dramatically between 6.2 and 9.8, making it impossible to stably ensure that the chlorine disinfection CT value is met. The absence of an anaerobic tank leads to COD load shocks to the aerobic system, reducing dissolved oxygen (DO) to 0.5 mg/L and causing a 70% decrease in nitrifying bacteria activity within 48 h. These failures continuously amplify public health risks (Niu et al., 2025). A study by Niu et al. on 11 hospitals in China confirmed this pattern. The scholars reported that 34% of the facilities lacked screens or equalization tanks, with influent pH values fluctuating between 6.9 and 9.1 and fecal coliform concentrations reaching 6.8–7.3 log CFU/100 mL. Hospitals without anaerobic tanks had only 0.6 mg/L DO in the aerobic zone, with a 68% decrease in nitrifying bacterial activity within 48 h. Moreover, debris entangling aerators created anoxic zones.

In addition, small-scale medical facilities face a severe “cost-benefit” imbalance and risk-shifting problems, resulting in a chronically underfunded wastewater infrastructure (Mousel et al., 2021). Considering rural-serving small clinics with fewer than 50 beds as an example, the investment per ton of water for their support of decentralized wastewater treatment systems is approximately $1,200–$1,400. If compliant technologies such as ultrafiltration membrane bioreactors (UF-MBR) are adopted, the initial equipment and installation costs can account for more than 40% of the facility’s annual budget (Lansing et al., 2017). This imbalance creates a typical vicious fund-site cycle: limited profits are insufficient to support the construction of standard treatment facilities occupying 3–5 square meters, whereas land constraints further narrow the adaptability of low-cost technologies (Pavan et al., 2007).

### 4.2 Mismatch between design capacity and actual demand

Building on the previous analysis of the chain-breaking risks caused by the absence of facilities, another key issue emerges: why do some seemingly fully constructed wastewater treatment systems still fail frequently. The core problem is not whether the system units “exist” but whether they are “adequate.” Over the past three decades, small medical institutions worldwide have generally adopted a “one-time blueprint” design approach. This approach determines the treatment load on the basis of the number of beds and outpatient visits in a benchmark year without further adjustment. However, medical demand, which is influenced by factors such as pandemics, aging populations, and the expansion of health insurance, has shown a continuously rising exponential curve. The contradiction between static design and dynamic demand has evolved from a mild crack to a systemic collapse.

A multicenter study of 16 general hospitals in Thailand described the operational crisis in intensive care units (ICUs) as a 120–133% overload, which essentially stems from a systemic imbalance between the non-linear growth of medical demand and static bed design (Abhicharttibutra et al., 2018). The study’s follow-up data revealed that from 2015 to 2019, the demand for hospital beds maintained a steady annual growth rate of 2.3%, which could be normally accommodated by the traditional static design; however, after the outbreak of the pandemic in 2020, the demand curve jumped in a stepped manner—within just 2 months, the demand for inpatient care for respiratory diseases increased by 280%, and the bed occupancy rate in some departments exceeded 133%, far surpassing the original design capacity. This dynamic characteristic of “stable accumulation-sudden surge” is universal worldwide. A survey of 183 countries conducted by the World Health Organization (WHO) confirmed that even in high-income regions (with an average of 402.32 hospital beds per 100,000 people), 42% of hospitals still had a bed occupancy rate exceeding 120% during the peak of the pandemic, fully exposing the rigid constraints of static design (Chen et al., 2017).

A 2024 Brazilian study utilized geographic information systems (GIS) to determine that hospital wastewater volumes increased by 65% over a decade, while the availability of developed land stagnated at zero growth (Lyu et al., 2018). Moreover, an invisible surge has been easily overlooked. Specifically, after outpatient infusion rooms were converted into inpatient wards, the bed turnover rate increased by 22%, resulting in a proportional increase in wastewater production. However, the design load was not updated accordingly to reflect these changes. Such “function-change surges” are typically ignored by traditional population-to-bed models. To address this gap, a dynamic “service intensity coefficient (SIC)” should be introduced. This coefficient would enable real-time correction of treatment loads and align them accurately with actual demand.

The consecutive collapse cases of three municipal sewage pumping stations in Kisumu County, Kenya, have profoundly exposed the inherent flaws of the centralized pumping model in small-scale medical scenarios. Medical sewage from 5 primary hospitals and 12 clinics in this region is connected to a centralized pipe network, which relies on pumping stations to transport it to a treatment plant 15 kms away. Between 2022 and 2023, the annual sewage volume increased by 9.3% because of the increase in medical demand, resulting in pumping stations operating in a long-term overloaded operation state. These stations eventually fail in sequence because of motor burnout and pipeline blockage (Omohwovo, 2024). More critically, the centralized system lacks a redundant design: a single unit failure directly leads to the shutdown of the entire system, and no backup power supply is available, resulting in 48 consecutive hours of direct sewage discharge. Medical wastewater containing antibiotics and pathogenic microorganisms flows directly into the upper tributaries of Lake Victoria. Since the natural dilution capacity of the downstream ecologically sensitive wetland area is only 0.8 times the standard, which is far lower than the 3.2-fold dilution requirement for municipal sewage, the concentration of *Escherichia coli* in the water exceeded the standard by 12-fold; the incidence of acute gastroenteritis in the coastal communities increased by 27% in the short term.

### 4.3 Institutional gap amplifying technological gaps

The failure cases of 5 small-scale wastewater treatment plants in rural areas of Cochabamba, Bolivia, are often simplistically attributed to wrong technology selection, but they are a concentrated reflection of systemic institutional deficiencies. The design of 3 treatment plants completely ignored the local annual population growth rate of 3.2% and used static data from 2010 to calculate the treatment load. Owing to the lack of a special assessment mechanism, the regulatory authorities failed to conduct onsite O&M evaluations for 4 consecutive years. The rate of combined rainwater and sewage connection reached 45%, yet there was no clear responsible entity to promote pipe network rectification (Cossio et al., 2018). This phenomenon accurately confirmed the “technology-institution coupling gap” theory: when an institution fails to provide dynamic adaptation (such as feedback on population growth), regulatory constraints (such as regular evaluations), and supporting guarantees (such as pipe network maintenance), even technological facilities that comply with standards degrade along a low-efficiency path because of the lack of external support, and they eventually fall into a vicious cycle of “operation failure-fault accumulation-complete paralysis” (Cossio et al., 2018).

### 4.4 Equipment aging and operation and maintenance management failure

Once the “mismatch between design capacity and demand” pushes the system to the overload threshold, equipment aging and operational maintenance mismanagement emerge as other critical influencing factors. If design mismatch is a “congenital flaw” of the system, equipment aging and operational maintenance failure constitute “acquired defects,” and their combination can gradually increase chronic wear-and-tear risks into gaps that trigger acute malfunctions.

**FIGURE 3:**
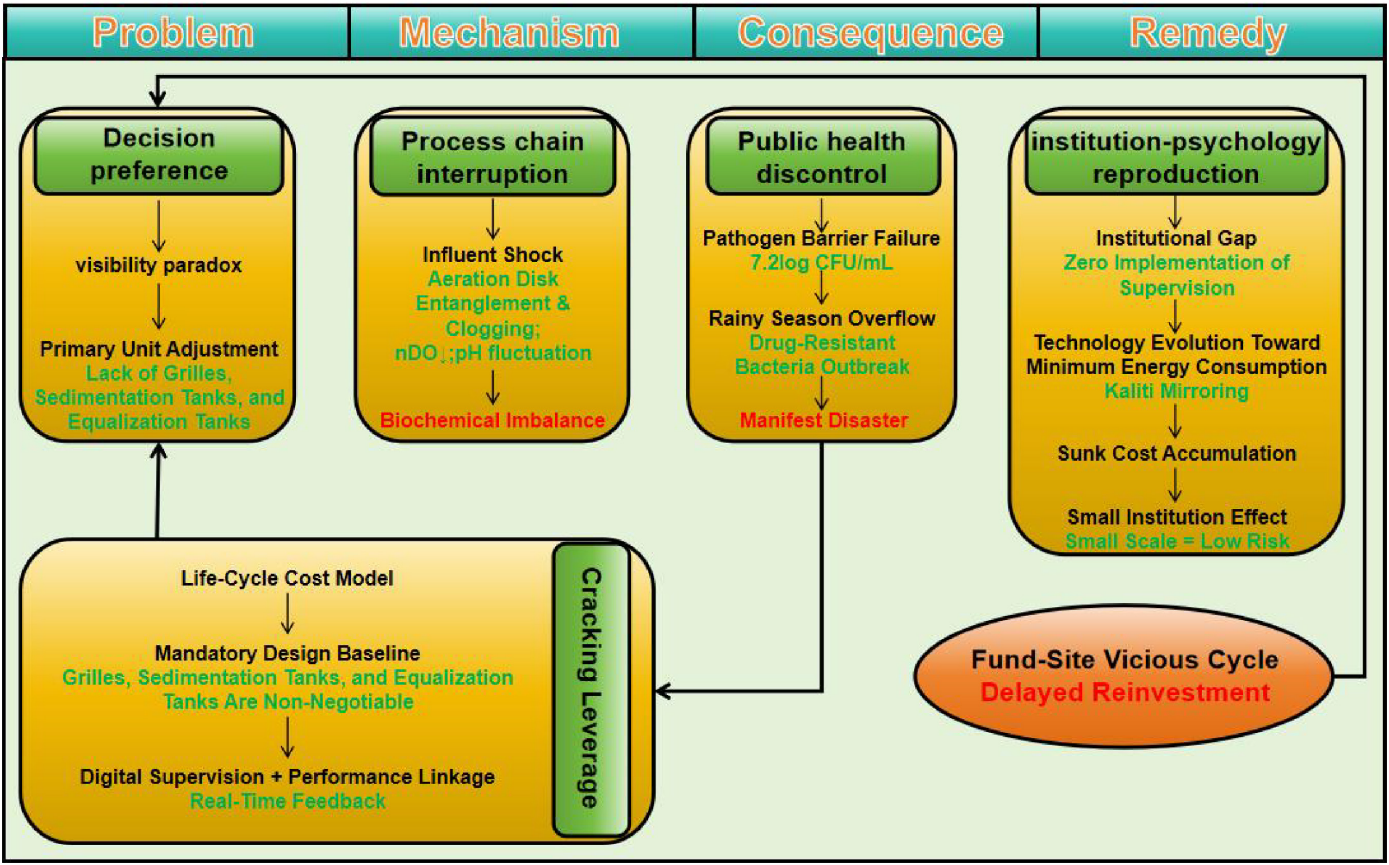
Analysis of sewage system issues from decision-making to consequences and remedies.

The logical chain of this process is illustrated in Figure 3. The “visibility paradox” at the decision-making end (e.g., streamlining of basic units such as screens and sedimentation tanks due to non-quantifiable value) forms the starting link. Subsequent interruptions (such as influent shocks and aerator clogging) gradually lead to consequences such as biochemical system imbalance and pathogen barrier failure. Moreover, equipment aging under mismanaged maintenance (e.g., membrane module damage and pump body corrosion) accelerates the deterioration of this chain, ultimately forming a vicious fund-site cycle and even triggering overt incidents such as rainy-season overflow and bacterial blooms.

In this section, the evolution process and core issues are analyzed on the basis of actual onsite operation scenarios.

Figure 3 illustrates the full causal chain of sewage system issues: decision preferences (e.g., the visibility paradox and removal of primary units such as grilles) lead to process chain interruptions and biochemical imbalance, which escalate into public health disasters (e.g., pathogen barrier failure and drug-resistant bacterial outbreaks). These issues are reinforced by institutional and psychological factors, forming a vicious fund-site cycle of delayed reinvestment. Proposed remedies include a life-cycle cost model, mandatory design baselines, and digital supervision to break the cycle.

Equipment aging is not a failure of a single component but rather cumulative multidimensional microscopic wear throughout the system. Membrane breakage or poor maintenance in the membrane bioreactor (MBR) process can impair the retention of antibiotics and their metabolites, potentially leading to rerelease of pollutants that becomes a hidden breakpoint for declining treatment stability (Paulus et al., 2019). For pumps and pipelines, long-term exposure to sodium hypochlorite disinfection residues and drug metabolites in medical wastewater causes galvanic corrosion and chemical erosion, leading to aging and leakage of pump seals and thinning of pipeline walls. Their leakage risk is 3 times greater than that of new equipment. In terms of the programmable logic controller (PLC) control system, the high-humidity environment and corrosive gases (such as hydrogen sulfide) in the wastewater treatment room result in capacitor breakdown and line aging, with a control accuracy drift, making it impossible to precisely regulate chemical dosage and aeration intensity (Zhang et al., 2018).

A special O&M survey of 28 primary hospitals in Maharashtra, India, revealed that 72% of the control cabinets for wastewater treatment systems have been in service for more than 15 years, becoming the core source of systemic failure. These aging devices generally have three major hidden dangers: (1) due to long-term arc ablation and environmental corrosion, the surface oxide layer on a relay contact surface reaches a thickness of 0.1–0.3 mm, and the contact resistance is 5–8 times greater than that of new equipment; (2) capacitor aging in frequency converters causes an output frequency drift of ± 9%, which fails to accurately match the aeration demand; and (3) 45% of the devices are not equipped with residual current protection devices, making their electric leakage risk 4 times greater than that of compliant equipment (Leiva et al., 2024).

The greatest threat of electrical system aging lies in the “chain amplification of hidden faults.” Relay contact adhesion is the most typical hidden hazard. A case study of a community hospital in India revealed that the relay contacts in the aeration system control cabinet adhered because of ablation, causing the blower to operate continuously at full speed. As a result, the DO concentration in the bioreactor decreased sharply from 2.5 to 0.4 mg/L within 2 h, the nitrifying bacterial activity decreased by 65%, and the ammonia nitrogen removal rate decreased from the designed 82 to 28%. Moreover, there is no obvious alarm in the early stage of such faults—the PLC control system fails to detect the “equipment overload signal” and displays only “normal operation.” O&M personnel do not notice the abnormality until the effluent ammonia nitrogen exceeds the standard, and they miss the optimal intervention opportunity (Leiva et al., 2024).

Quantitative analysis from the 2025 Medical Wastewater O&M Study revealed that for every 10% increase in the “preventive maintenance deficit,” the probability of equipment failure increases by 18% (Zhang et al., 2021). When maintenance investment falls below 70% of the original equipment manufacturer (OEM) recommended level, risk increases exponentially. Conversely, when investment exceeds 90%, risk reduction plateaus. Thus, an optimal maintenance zone exists to balance cost and risk. For example, in Fijian medical institutions, the frequent failures of aerators are superficially attributed to budget shortages (Farrell et al., 2015); however, the underlying cause is fragmented maintenance outsourcing. Multiple suppliers competing on low prices lead to decreased spare part quality and extended maintenance cycles. To address this issue, scholars suggested the establishment of regional integrated maintenance centers. These centers achieve unified spare part procurement, unified operation and maintenance personnel training, and centralized fault scheduling, thereby improving maintenance efficiency.

### 4.5 Odor and noise triggering community conflicts

A survey conducted by a sociology team at the University of Fiji revealed that the unacceptability rate of odors emanating from wastewater treatment systems in healthcare facilities is alarmingly high, reaching 72% (Farrell et al., 2015). In today’s digital age, where social media amplifies information exponentially, a single odor event can go viral, thereby damaging the social credibility of a hospital. Thus, odor control transcends being merely a technical issue and has become a pivotal aspect of public relations management. Echoing this concern, a related study in 2024 revealed that treatment facilities with a sludge discharge cycle exceeding 30 days experience a pump failure rate that is 3.4 times higher than those with a cycle of less than 7 days (Liu Y. et al., 2025).

### 4.6 Lack of professional technical personnel and insufficient training

When equipment aging and failure push the system to the brink of collapse, it is not concrete and steel that truly determines whether the system can stabilize but rather its human element. In this section, we shift the focus from mechanical wear and tear to the systemic deficit in human capital, which is characterized by low certification rates, short training durations, and high attrition rates. We explore how these technical gaps can be amplified into governance fractures, ultimately evolving into compliance risks and crises of public trust.

A global assessment in 2024 revealed that more than 50% of institutions lack dedicated wastewater operators, with certification rates below 10% (Liang and Yue, 2021; Serafeim et al., 2024). The authors suggest that behind these numbers lies the issue of “occupational attractiveness,” namely, low wages for grassroots positions, narrow career advancement pathways, and poor social recognition, all of which drive talented young people away from this field. Through interviews, it was found that existing staff lack training in biological treatment processes. Operators typically equate “sludge age” with “sludge discharge cycle” and simplify “DO setpoint” to “by feel.” This knowledge gap not only affects operation but also hinders communication with regulatory authorities—since they cannot demonstrate compliance in professional terms, they can only passively accept fines.

The UK Healthcare Facility Operation and Maintenance Report indicated that for every 10% increase in the certification rate of wastewater operators, the probability of fecal coliform exceedance in effluent decreases by 7% (Embleton et al., 2023). Scholars further constructed a “certification-performance” elasticity curve: when the certification rate exceeds 60%, the probability of effluent exceedance approaches zero; when the certification rate is below 20%, the exceedance probability increases exponentially. A related study in Brazil confirmed the importance of the timeliness of training—individuals who received only 2 days of onboarding training had their professional knowledge accuracy rate decrease to 38% after 3 months. In light of this issue, the adoption of a “microcertification” training model is recommended: a 2-h online theoretical course combined with on-site practical assessment every quarter ensures that the skills of certified personnel are continuously updated and that knowledge decay is avoided (Alves et al., 2021; Boldrin et al., 2022).

A tracking study of medical wastewater treatment institutions in Kenya revealed an annual staff turnover rate of 30% in this field. New employees require 6–8 weeks to work independently, during which the stability of system operation significantly declines (Jiang L. et al., 2023; Lee et al., 2024; Tanui et al., 2025). To address this issue, a dual-track inheritance system of “mentorship + cloud-based knowledge” can be established. Experienced senior staff members can act as mentors to train new employees. Moreover, all the operational parameters, troubleshooting records, and equipment maintenance logs can be uploaded to the cloud to form a searchable technical knowledge graph. This approach can help avoid the discontinuity of technical experience due to staff turnover. Related studies (Jeske and Gallert, 2021; Maragkaki et al., 2018; Yu et al., 2016) have noted that even if the treatment system design is perfect, it will fail without continuous education support. In fact, continuous education is not only a means of skill renewal but also a way to rebuild professional dignity. When operators can communicate efficiently with engineers in professional terms, their professional identity and work enthusiasm are significantly enhanced. This phenomenon, in turn, can reduce their intention to leave.

Globally, more than 50% of institutions are struggling with a shortage of staff, Brazil sees a mere 38% knowledge retention rate 2 days after training, and Kenya has an annual staff turnover rate of 30%. Such human capital deficits have spawned a vicious cycle: low position attractiveness leads to subpar training quality, which in turn drives high turnover, resulting in poor operational performance and elevated penalty risks and further eroding attractiveness. To break this cycle, it is imperative to integrate microcertification training into quarterly operation and maintenance workflows, embed a mentorship mechanism into job handover standards, and leverage cloud-based knowledge bases as a technical support system for onsite operators. Only through these measures can the wastewater treatment stations of small medical institutions transform from unmanned operation to specialized management and from passive penalty acceptance to active regulatory compliance, ultimately realizing the coevolution of technical and human capital systems. In summary, the operational and management challenges of sewage systems and their core logical analysis based on the analyzed cases are synthesized in Figure 3.

**FIGURE 4:**
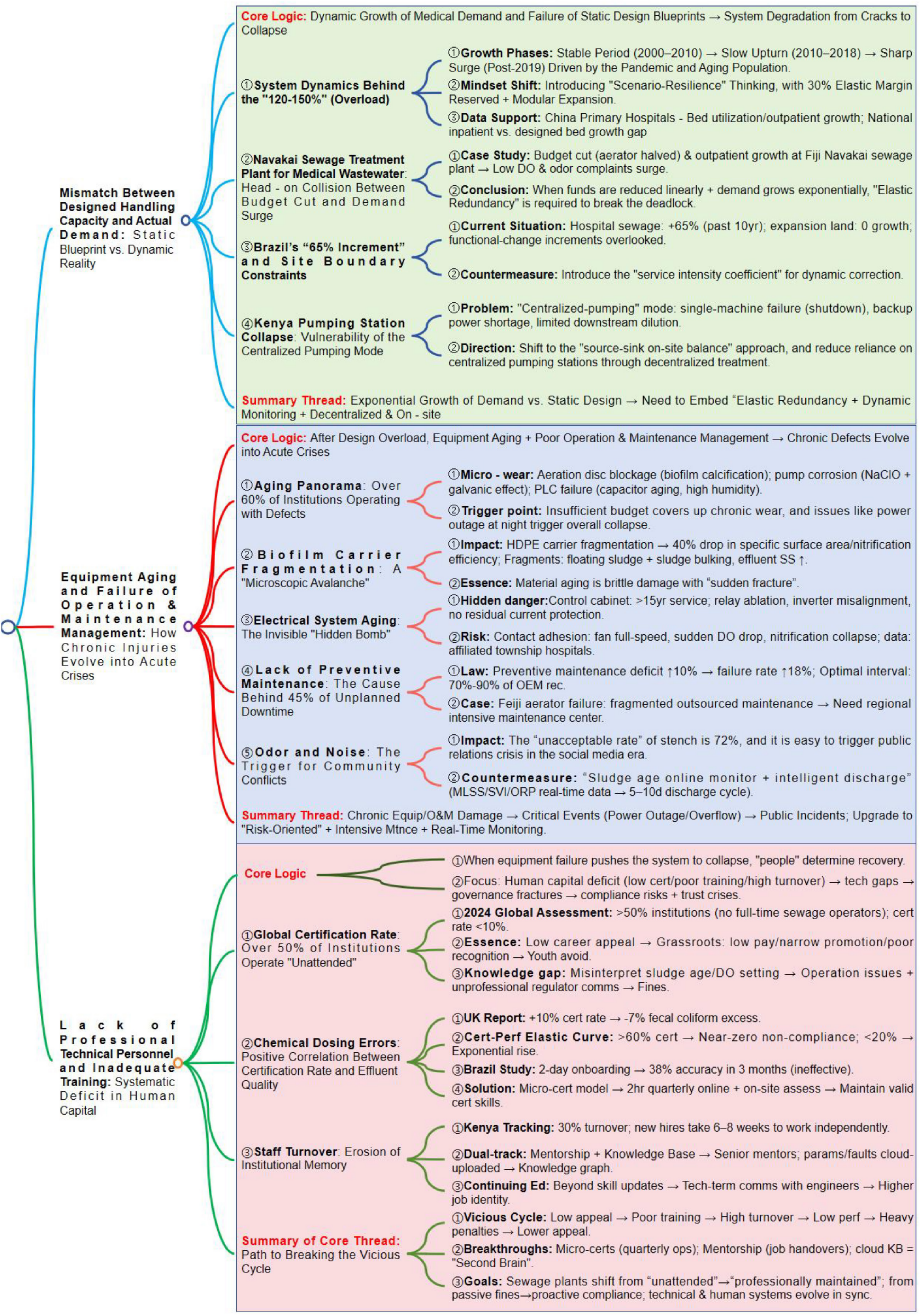
Analysis of challenges and core logics in sewage system operation and management.

In Figure 4, we systematically analyze the core challenges and causal logics in sewage system operation and management, covering three interconnected dimensions: the mismatch between static design blueprints and dynamic medical demand growth, the evolution of chronic equipment aging and poor maintenance into acute crises, and the vicious cycle of human capital deficits (insufficient training and high turnover) and its mitigation strategies. We summarize the core threads: integrating elastic redundancy and dynamic monitoring to address demand surges, adopting risk-oriented intensive maintenance to manage chronic equipment damage, and breaking the vicious cycle of human capital through microcertification, mentorship, and cloud-based knowledge bases to achieve sustainable system operation.

## 5 Impact of residual antibiotics in hospital wastewater on the environment

### 5.1 Impact on aquatic organisms

**FIGURE 5:**
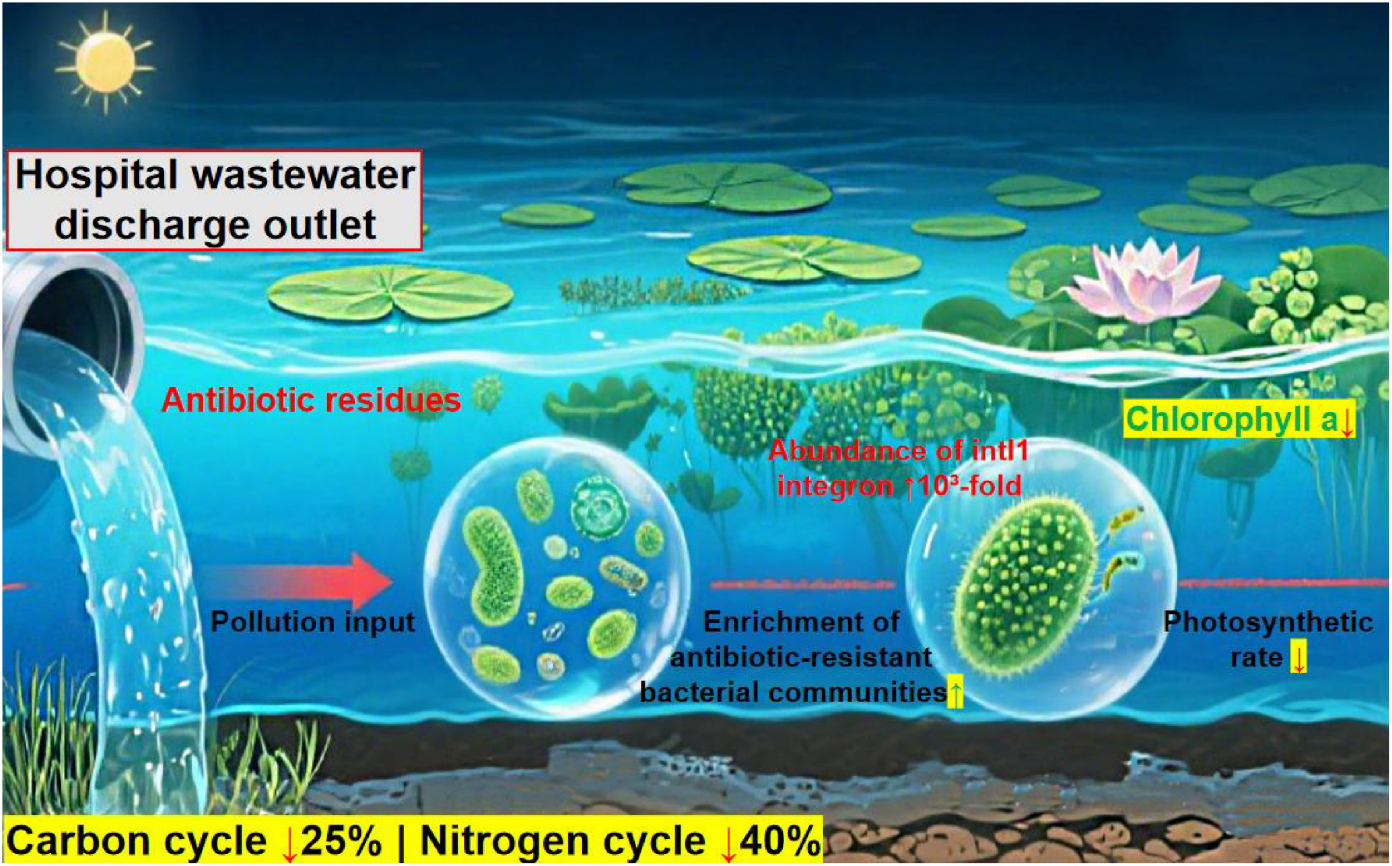
Impacts of hospital wastewater discharge on aquatic ecosystem processes and microbial communities.

The entry of antibiotics into hospital wastewater is a key pathway through which antibiotics enter the environment. Residual antibiotics (e.g., β-lactams, sulfonamides, and fluoroquinolones) are incompletely degraded in treatment systems and flow into natural water bodies. As shown in Figure 5, long-term low-concentration exposure disrupts aquatic ecosystems: it increases the abundance of antibiotic-resistant bacterial communities, increases the abundance of resistance integrons (e.g., *intI1*), reduces carbon/nitrogen cycle efficiency by 25%/40%, and impacts indicators such as chlorophyll a. This interference spans microbial communities to higher organisms, thus negatively affecting ecological balance (Akhter et al., 2024; Langbehn et al., 2021; Yang et al., 2021).

Figure 5 shows that hospital wastewater discharge introduces antibiotic residues, which enrich antibiotic-resistant bacterial communities and increase *intI1* integron abundance by 10^3^-fold. These discharges reduce carbon and nitrogen cycle efficiency by 25 and 40%, respectively, and suppress primary productivity indicators such as chlorophyll a and the photosynthetic rate.

#### 5.1.1 Selective interference with microbial communities and the spread of antibiotic resistance

Microbial communities play crucial roles in driving the cycling of carbon, nitrogen, phosphorus, and other elements in aquatic environments (Kaszubinski et al., 2022). When exposed to low concentrations of antibiotics over extended periods, these communities face significant selective pressures, leading to structural imbalances and functional disorders. This phenomenon is particularly evident in the waters and sediments downstream of hospital wastewater discharge outlets, where the impact is more pronounced (Li et al., 2021; Emara et al., 2023). Antibiotics exert selective pressure that enriches antibiotic-resistant strains. For instance, the abundance of *Vibrio* spp. can increase 10- to 100-fold downstream compared with upstream, with a substantial increase in the proportion of strains carrying β-lactamase genes (such as *bla*_*OXA*_) and tetracycline resistance genes (such as *tetE*) (Pavón et al., 2022; Esposito et al., 2024). This selective enrichment not only alters the community composition but also reduces metabolic activity. In water contaminated with common antibiotics such as quinolones, the efficiency of microbial-mediated carbon cycling decreases, and the rates of key nitrogen cycling processes, such as ammonification and nitrification, decrease. This phenomenon weakens the self-purification capacity of the water, is observed in both single- and mixed-antibiotic exposure scenarios, and poses a significant ecological risk in aquatic environments such as reservoirs and recreational waters (Huang et al., 2020; Nappier et al., 2020).

Continuous exposure to antibiotics facilitates the horizontal transfer of ARGs among bacteria through mobile genetic elements (MGEs), such as plasmids, transposons, and integrons. This process endows pathogenic bacteria in the natural environment, such as *E. coli*, with antibiotic resistance (Nappier et al., 2020; Pavón et al., 2022; Tuvo et al., 2023). For example, in estuarine sediments contaminated by hospital wastewater, the abundance of class 1 integrons is positively correlated with sulfonamide antibiotic concentrations. The sul1 gene they carry can be transferred to enteric pathogens via conjugation, thereby increasing the likelihood of health risks posed by environmental pathogens (Nappier et al., 2020; Zhang et al., 2024). The predominance of resistant bacteria disrupts the natural balance of microbial communities and exacerbates ecosystem instability (Zhang T. et al., 2025).

#### 5.1.2 Inhibition of algal and zooplankton growth

Antibiotics can influence primary producers in aquatic ecosystems (e.g., algae) through the food chain. Algae are highly susceptible to antibiotic interference, and their growth and metabolism are crucial for the energy supply of ecosystems (Abdelfattah et al., 2023). For instance, sulfonamides (e.g., sulfamethoxazole) and fluoroquinolones (e.g., ciprofloxacin) can inhibit the activity of key enzymes involved in algal photosynthesis, such as Rubisco and PSII reaction center proteins, thereby reducing photosynthetic efficiency (Huang et al., 2020; Wan et al., 2024). Experiments have demonstrated that in artificial seawater aquaculture wastewater, when the concentration of sulfamethoxazole (SMX) reaches 100 ng/L, the growth of *Chlorella* sp. biofilms is affected. This phenomenon results in reduced chlorophyll a content and impaired photosynthetic activity, with related metabolic processes being inhibited. This concentration is consistent with the residual levels found in actual seawater aquaculture wastewater, and it has adverse effects on the physiological functions of *Chlorella* sp. (Wang M. et al., 2022). Similarly, when the concentration of ciprofloxacin reaches 200 ng/L, the cell division rate of *Scenedesmus obliquus* decreases by 25%. In addition, abnormal phenomena such as thickened cell walls and disintegrated chloroplasts occur (Hejna et al., 2022; Wan et al., 2024).

The inhibition of algal growth has a direct effect on the zooplankton that feed on them, such as cladocerans and copepods. In water contaminated with antibiotics, the food supply for zooplankton is diminished. This process results in a 10–15% decrease in their survival rates and a 20–30% reduction in their reproductive capacity (Nappier et al., 2020). Tetracycline exerts a cascading effect on aquatic ecosystems. This effect initially inhibits the photosynthetic process of *Scenedesmus obliquus*, thereby reducing algal biomass. This phenomenon, in turn, affects the survival and reproduction of *Daphnia magna*. When the concentration of tetracycline in the environment reaches 100 ng/L, the hatching rate of *Daphnia magna* larvae decreases by 12%, and the adult lifespan is shortened by 15%. This process is characterized by slow growth, a shortened reproductive period, and a reduced survival rate of newly hatched larvae. These findings clearly demonstrate that by interfering with primary producers, tetracycline indirectly hinders the growth and development of zooplankton (Chen et al., 2022; Nappier et al., 2020). A reduction in the number of primary consumers affects the energy acquisition of organisms at higher trophic levels in the food chain, thereby triggering ecological cascades.

#### 5.1.3 Physiological and immune damage to higher-order aquatic organisms such as fish

As higher-order consumers in aquatic ecosystems, fish are highly susceptible to the interference of residual antibiotics with their physiology, immunity, and reproduction. Long-term exposure to environmentally relevant concentrations (0.01–0.1 mg/L) of antibiotic mixtures, such as sulfamethoxazole combined with clarithromycin, significantly suppresses the immune defense system of fish (Ballash et al., 2022). For example, after 2 weeks of exposure, the bacterial load in the gut and kidneys of *Danio rerio* increased 2–3-fold. Additionally, 40–60% downregulation of the expression of key genes of the complement system (*c3a*, *c4*, and *c9*) was detected. This detection led to increased susceptibility to pathogens, such as spring viremia of carp virus, and a 35% increase in mortality (Pereiro et al., 2022). Moreover, fluoroquinolone antibiotics induced abnormal expression of cytochrome P450 enzymes in the liver of fish. This process led to disordered drug metabolism and increased oxidative stress levels, characterized by a 25% increase in malondialdehyde content and a 30% decrease in superoxide dismutase (SOD) activity (Topić Popović et al., 2023).

Antibiotics disrupt the endocrine and reproductive functions of fish. Fluoroquinolones and tetracyclines inhibit the activity of gonadal steroidogenic enzymes such as 3β-HSD and 17β-HSD, thereby reducing the synthesis of sex hormones such as estradiol and testosterone (Huang et al., 2020; Nappier et al., 2020). Studies have shown that *Oreochromis niloticus* exposed to 500 ng/L ciprofloxacin exhibit a 10–30% reduction in egg production in females and a 25% decrease in vitellogenin (VTG) content. In males, sperm motility decreases by 20–40%, and the rate of malformations increases by 15% (Aranaga et al., 2022). This decline in fish reproductive capacity threatens population sustainability and affects the structural stability of aquatic ecosystems (Bojarski et al., 2020).

Hospital wastewater often contains residual antibiotics, which pose a significant threat to aquatic life. These antibiotics affect organisms at various levels of the food chain through multiple pathways. Antibiotics disrupt the natural cycles of matter and the flow of energy within ecosystems. Moreover, antibiotics increase ecological risk because of the spread of antibiotic resistance genes and the resulting decline in certain populations. To address this pressing issue, it is crucial to address the problem at its source. Upgrading wastewater treatment technologies and enhancing environmental monitoring are essential steps for effectively controlling the release of antibiotics into the environment and mitigating severe ecological consequences.

### 5.2 Ecological impacts of antibiotic residues on aquatic ecosystems

When residual antibiotics from hospital wastewater enter water bodies, they pose a dual threat. These antibiotics not only directly affect aquatic organisms but also undermine the structural integrity and functional stability of aquatic ecosystems. This phenomenon occurs through a cascade of effects: altering community structures, disturbing biological behaviors, and interacting with other pollutants. Moreover, these ecological disturbances are often long term and irreversible, leading to profound and lasting impacts on the health and resilience of aquatic environments.

#### 5.2.1 Imbalance in community structure: dominance of resistant bacteria and disruption of niche competition

One of the most significant impacts of antibiotic residues on aquatic ecosystems is the promotion of antibiotic-resistant bacteria to dominant populations through selective pressure, which disrupts the niche balance of microbial communities (Akhter et al., 2024). This phenomenon is particularly pronounced in sediments and water near hospital wastewater discharge outlets. For example, the abundance of resistant *Aeromonas hydrophila* can account for more than 30% of the total bacterial population in these areas.

These bacteria not only carry multiple resistance genes (such as the β-lactam resistance gene *bla*_*OXA*_ and the tetracycline resistance gene *tetE*) (Dubey et al., 2022; Wu et al., 2023) but also secrete antimicrobial substances (such as aerolysin). This phenomenon significantly inhibits the activity of nitrifying bacteria (such as *Nitrosomonas* and *Nitrobacter*). These bacteria are key players in the nitrogen cycle of water bodies and are responsible for converting ammonia nitrogen to nitrate (Guerra et al., 2022). When nitrifying bacteria are inhibited, the concentration of ammonia nitrogen in the water can increase by 2–3 times. This increase leads to water quality deterioration and subsequently affects the survival environment of algae and aquatic animals (Jiao et al., 2011).

The disruption of niche competition triggered by antibiotic-resistant bacteria is far-reaching. The overproliferation of these resistant strains can outcompete that of other functional microorganisms. For instance, *Pseudomonas*, which plays a vital role in degrading organic matter, and *Desulfovibrio*, a key participant in the sulfur cycle, can be crowded out. This process leads to a simplification of the metabolic functions of the microbial community (Zakhour et al., 2024). In estuarine areas contaminated with antibiotics, the consequences are striking. The abundance of microorganisms involved in organic matter degradation decreases by 40%. This decline results in a 25–30% reduction in the removal efficiency of COD in the water, which further exacerbates the barriers to material cycling within the ecosystem (Zheng et al., 2021). Fundamentally, this community structure imbalance, dominated by resistant bacteria, is the result of antibiotics selectively enabling the “survival of the fittest” among microorganisms. However, this process often comes at the expense of ecosystem multifunctionality.

#### 5.2.2 Behavioral disturbances: cascading risks from individual survival to population sustainability

While residual antibiotics may not directly cause the death of aquatic organisms, they pose a significant threat to population sustainability and food chain stability by altering critical behaviors such as predation, predator avoidance, and reproduction. These disruptions can be broadly categorized into the following two major dimensions: animals and plants.

As key consumers in aquatic ecosystems, fish are highly susceptible to low concentrations of antibiotics. The core mechanisms underlying this vulnerability stem from the combined effects of neurotoxicity, oxidative stress, and immunosuppression. Tetracycline antibiotics can inhibit the activity of acetylcholinesterase in fish, thereby impairing neural transmission (Nunes et al., 2015). This inhibition results in delayed predator avoidance responses in freshwater fish, such as *Gambusia holbrooki*, thereby increasing their predation risk. Moreover, the energy metabolism disorders and muscle function damage caused by antibiotics can shorten swimming distances and reduce activity intensity (Lee et al., 2024; Melvin and Wilson, 2013; Pomati et al., 2006; Saaristo et al., 2018), further weakening their survival ability. Antibiotics can downregulate the expression of fish complement system genes (such as *c3a* and *c4*) (Bera et al., 2020; Hasan et al., 2024), thereby reducing immune perception. This downregulation leads to disordered recognition and escape behaviors in response to pathogens. Under biotic stress, it is significantly more difficult for fish to mount effective responses. The olfactory system of migratory fish is crucial for recognizing chemical signals in spawning grounds. However, fluoroquinolone antibiotics may damage olfactory epithelial cell signaling (Legradi et al., 2018; Vaudin et al., 2022), thereby reducing chemotactic responses to specific signals. This damage can lead to errors in migration or spawning ground localization, indirectly lowering population reproductive success (Brodin et al., 2014).

As primary producers, aquatic plants play vital ecological roles, such as stabilizing sediments, purifying water, and providing habitats. These roles are significantly affected by antibiotics, with different life forms showing varied responses (Gholipour et al., 2024; Langbehn et al., 2021; Ohore et al., 2024; Sharma et al., 2021; Zhang et al., 2022). When fully immersed in water, these plants experience full exposure to antibiotics. Tetracycline and ciprofloxacin can inhibit photosynthesis-related enzyme activity and damage cell structures, reducing growth rates by 30–50% and biomass by a similar margin (Sharma et al., 2021). This finding weakens their ability to stabilize sediments and purify water, leading to habitat fragmentation and threatening the animal populations that depend on them. When roots directly absorb antibiotics from water, sulfonamides can interfere with nitrogen metabolism, reducing the chlorophyll content by 20–40% and decreasing photosynthetic efficiency (Gholipour et al., 2024; Leister et al., 2022). Imbalanced growth may lead to overpopulation (surface shading-induced algal blooms) or a decline (reduced pollutant absorption capacity), disrupting the aquatic nutrient cycling balance. With roots in sediments and shoots exposed to air, these plants show a partial exposure response. Quinolone antibiotics in sediments shorten root lengths by 15–25%, whereas antibiotics in water interfere with shoot transpiration (Feng et al., 2025; Leister et al., 2022). Although this process does not directly threaten individual survival, it weakens their ability to stabilize shores, protect banks, and provide habitats for birds, indirectly leading to community structure disintegration.

#### 5.2.3 Synergistic effects with microplastics: amplification of ecological risks

Antibiotics and microplastics, both ubiquitous in aquatic environments, can interact to significantly amplify ecological risks at the individual, population, and ecosystem levels. By acting as carriers, microplastics can increase the bioavailability of antibiotics (Zhao et al., 2023). For example, combined exposure to nanoplastics and tetracycline antibiotics exacerbates the abnormal expression levels of antioxidant and immune-related genes in *Danio rerio*. This process not only promotes the distribution of antibiotics within fish tissues but also leads to pathological damage to intestinal tissues (Wu et al., 2025; Yu et al., 2024). Similarly, after 8 weeks of exposure to a mixture of polyvinyl chloride (PVC) microplastics and oxytetracycline/sulfamethoxazole, common carp show significant enrichment of potentially pathogenic bacteria such as *Enterobacteriaceae* and *Acinetobacter* in their intestines (Li et al., 2024).

Coexposure to microplastics and clarithromycin can significantly inhibit the feeding ability of Daphnia magna. This exposure has additive effects on SOD activity and slight synergistic effects on catalase activity (Trinh et al., 2024). These disruptions in survival and toxicological responses can affect population reproduction and growth. In shrimp aquaculture systems, microplastics can promote biofilm formation, accelerating the horizontal transfer of ARGs and the proliferation of pathogenic bacteria. This phenomenon leads to the formation of an “ARG-pathogen-microplastic pollution complex,” which increases the risk of multiple drug-resistant bacterial infections in shrimp (Páez-Osuna et al., 2024).

Microplastics can serve as vectors for microbial migration, facilitating the colonization, spread, and enrichment of ARGs in aquatic environments and sediments (Bank et al., 2020; Goswami et al., 2025). This process alters microbial community structure and function, thereby disrupting key ecosystem processes such as material cycling and energy flow.

By inducing community structure imbalances, disturbing biological behaviors, and interacting with microplastics, residual antibiotics in hospital wastewater cause multidimensional and cascading damage to aquatic ecosystems. This impact extends from microorganisms to higher-order organisms and from individual functions to system stability. This phenomenon highlights the urgency of controlling antibiotic residues at the source and of upgrading technologies to increase their removal from hospital wastewater.

## 6 Impact of hospital wastewater on microbial antibiotic resistance

**FIGURE 6:**
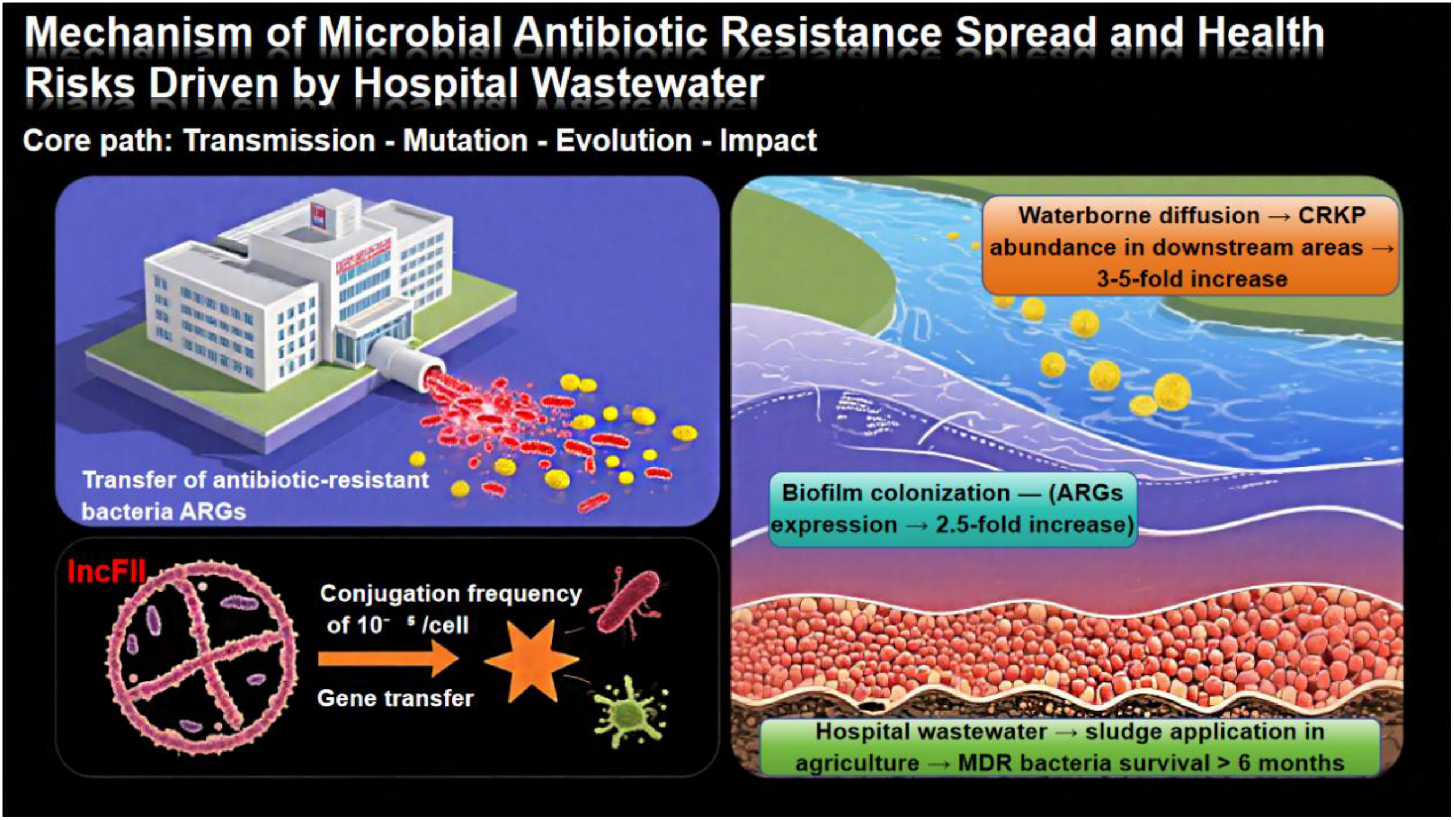
Mechanisms of the spread of microbial antibiotic resistance and health risks driven by hospital wastewater.

Hospital wastewater, a complex matrix laden with high concentrations of antibiotics, antibiotic-resistant bacteria (ARB), and ARGs, serves as a primary pathway for the spread of antibiotic-resistant microbes into the environment. As shown in Figure 6, this spread follows a “transmission-mutation-evolution-impact” core path: hospital wastewater releases ARB/ARGs that diffuse via water, colonize biofilms, and persist in soil, with plasmid-mediated gene transfer (e.g., *IncFII* plasmids) further facilitating resistance dissemination. Recent global studies have confirmed that these ARB and ARGs infiltrate ecosystems through water and soil routes, posing a significant threat to public health (Medugu et al., 2023; Mutuku et al., 2022).

Figure 6 depicts the core path of “transmission–mutation–evolution–impact:” hospital wastewater releases ARB and ARGs, which spread via waterborne diffusion and biofilm colonization. Plasmid-mediated gene transfer (e.g., *IncFII* plasmids, conjugation frequency of 10^–5^ per cell) accelerates dissemination, whereas MDR bacteria in sludge persist > >6 months after agricultural application.

### 6.1 Core characteristics of ARB and ARGs in hospital wastewater

ARBs in hospital wastewater are primarily *Enterobacteriaceae* but also include clinically important non-fermenting bacteria. The ARGs carried cover a variety of antibiotic classes, including β-lactams, aminoglycosides, and quinolones. The specific characteristics are as follows.

*K. pneumoniae*: This is the most frequently detected ARB in hospital wastewater and is associated mainly with the presence of carbapenemase genes. For example, a study by Liu et al. on wastewater from a tertiary hospital in Jilin revealed that 91.4% of CRKP strains carried *bla*_*KPC*_, 82.8% were multidrug resistant (MDR), and 17.2% were extensively drug resistant (XDR). Some ST11 strains carried high-virulence genes (*rmpA* and *iucA*), posing a dual risk of “high resistance + high virulence” (Liu et al., 2023; Martins et al., 2022). In hospital wastewater from the Eastern Cape of South Africa, the amoxicillin resistance rate of this species reached 86%, and it mainly carried tet, sul, and β-lactam ARGs (Okafor and Nwodo, 2023a).

*E. coli*: *E. coli* is present predominantly in the form of the ST131 and ST38 types. These strains carry extended-spectrum β-lactamase (ESBL) genes, such as *bla_*CTX*–*M*–15_* and *bla_*CTX*–*M*–27_*, and carbapenemase genes, such as *bla_*OXA*–244_*. A study by Grevskott et al. (2024) revealed that the *bla_*OXA*–244_* gene carried by ST38 *E. coli* from hospital wastewater was highly homologous to the gene sequences of clinically epidemic strains (with only 1–18 SNP differences), confirming that hospital wastewater is the source of its environmental spread. Moreover, this bacterium can transfer ARGs such as *tet(A)* and *strA*/*strB* via the *IncFIB* plasmid (Mutuku et al., 2022, Garba et al., 2023).

*Aeromonas* spp.: These gram-negative bacteria with strong environmental adaptability often carry multiple ARGs. For instance, Zhu et al. isolated a strain of *Aeromonas veronii* (HD6454) from hospital wastewater in Suzhou, which simultaneously carried bla_*KPC*–2_ (carbapenem resistance), mcr-3.17 (colistin resistance), and *tmexC3.2*-*tmexD3.3*-*toprJ1b* (tigecycline resistance), highlighting their role as ARG reservoirs (Yang et al., 2022; Zhu et al., 2023).

Other ARB: *Pseudomonas aeruginosa* carries *bla_*OXA*–21_* (β-lactam resistance) and *aacA3* (aminoglycoside resistance) (Okafor and Nwodo, 2023b); *Acinetobacter baumannii* ST2 type carries *bla_*OXA* –__58_*, with a detection rate of 12.5% in hospital wastewater from a hospital in Zhejiang (Gu D. et al., 2024). *Staphylococcus* spp. exhibit methicillin resistance mediated by the *mecA* gene, with a detection rate reaching 30% in hospital wastewater (Velazquez-Meza et al., 2024).

The characteristics of ARB and antibiotics in hospital wastewater are closely associated with the clinical medication preferences of medical institutions and regional healthcare practices. Typical cases from hospitals worldwide further confirm the specificity of antibiotic-pathogen correlations (Table 2).

**TABLE 2:** Hospital wastewater antibiotics and core pathogenic bacteria.

Hospital	Country/region	Main types of antibiotics used	Core pathogens	References
A tertiary hospital in Jilin	China	β-lactams (predominantly carbapenems)	Carbapenem-resistant *Klebsiella pneumoniae* (CRKP)	Liu et al., 2023
A hospital in Suzhou	China	Carbapenems, colistin, tigecycline	*Aeromonas veronii*	Zhu et al., 2023
Hospitals in the Eastern Cape Province	South Africa	Amoxicillin, β-lactams, tetracyclines	*Klebsiella pneumoniae*, *Escherichia coli*	Okafor and Nwodo, 2023a
Hospitals in Southeastern Brazil	Brazil	β-lactams (predominantly cephalosporins)	*Escherichia coli*, *Klebsiella pneumoniae*	Batista et al., 2023
A hospital in Norway	Norway	Carbapenems (imipenem, meropenem)	*Escherichia coli* ST38	Grevskott et al., 2024
A hospital in Zhejiang	China	β-lactams	*Acinetobacter baumannii* ST2	(Gu D. et al., 2024)
Primary hospitals in Maharashtra	India	β-lactams, aminoglycosides	*Pseudomonas aeruginosa*	Anssour et al., 2016
A community hospital	India	Broad-spectrum antibiotics (specific types not specified)	*Klebsiella pneumoniae*, *Escherichia coli*	Leiva et al., 2024

### 6.2 Environmental dissemination of antibiotic resistance: pathways for bacteria and genes

Hospital wastewater facilitates the spread of antibiotic resistance into the environment via two primary pathways, direct diffusion and HGT, thereby establishing a transmission chain that extends from hospitals to wastewater treatment plants and ultimately to natural ecosystems. The specific routes are as follows.

#### 6.2.1 Direct diffusion pathways

The concentration of ARB in hospital wastewater is extremely high, with the concentration of CRKP reaching 10^3^–10^4^ CFU/mL. Even after treatment in wastewater treatment plants, the survival rate of CRKP in the effluent can reach 10–20%, causing the abundance of CRKP in receiving rivers to increase by 2–3 orders of magnitude compared with the background levels (Liu et al., 2023). For example, in the southeastern region of Brazil, untreated hospital wastewater discharged into urban rivers has increased the detection rates of *bla*_*KPC*_ and *bla_*CTX*–*M*_* in *E. coli* and *K. pneumoniae* in the river by 40–60% compared with upstream levels (Batista et al., 2023).

Soil and biofilm colonization: ARB can enter the soil through agricultural irrigation and sludge fertilization. The proportion of multidrug-resistant (MDR) bacteria in hospital wastewater sludge exceeds 70%, and strains such as *K. pneumoniae* ST11 and *E. coli* ST38 can survive in the soil for more than 6 months (Mutuku et al., 2022). Moreover, ARB easily adheres to the inner walls of pipelines and activated sludge flocs to form biofilms. The expression levels of ARGs within biofilms are 2–5 times greater than those in planktonic states, and their tolerance to disinfectants is increased, resulting in long-term sources of antibiotic resistance pollution (Martins et al., 2022).

Environmental ARB can contaminate crops and aquatic products. For instance, near the discharge outlets of hospital wastewater, the detection rate of *K. pneumoniae* in freshwater shrimp reaches 35%, with 80% of the *K. pneumoniae* carrying the *bla*_*KPC*_ gene (Velazquez-Meza et al., 2025). Human consumption of undercooked contaminated food can increase the risk of community-acquired infections.

#### 6.2.2 Horizontal gene transfer

The nutrient-rich environment of hospital wastewater provides ideal conditions for HGT, with mobile genetic elements (plasmids, integrons, and transposons) serving as the core vehicles for the interspecies spread of ARGs:

Plasmid mediation: *IncF*-type plasmids (e.g., *IncFII* and *IncFIB*) are most widely distributed in *Enterobacteriaceae*. In 91.4% of the CRKPs, the bla_*KPC*_ gene is located on the *IncFII* plasmid, which shares 92% sequence homology with the *IncFII* plasmid in *E. coli* and has a transfer frequency reaching 10^–5^ per recipient cell. *IncP-6*-type plasmids can carry bla_*KPC*–2_ for interspecies transfer, such as the same *IncP-6* plasmids found in *Citrobacter freundii* and *Klebsiella variicola* in Japanese hospital wastewater (Ota et al., 2022).

Class 1 integrons are detected in 61% of *E. coli* and *K. pneumoniae* in hospital wastewater; these integrons often carry gene cassettes such as *aadA5* (aminoglycoside resistance) and *dfrA17* (sulfonamide resistance) and are associated with the transposon Tn402 (Batista et al., 2023). The integrons can flexibly transfer ARGs to non-*Enterobacteriaceae* bacteria such as *P. aeruginosa* (Liu et al., 2023).

Transposons facilitate the transfer of ARGs through mechanisms such as “cut-and-paste” or “replication-and-paste.” For example, the transposon Tn6296, which carries the *bla_*KPC*_-2* gene, is commonly found in *K. pneumoniae*. Another example is Tn6855, which is associated with a cluster of resistance genes and has been inserted into the chromosome of an *OXA-244*-positive *E. coli* ST38 strain in Norwegian hospital wastewater. The presence of these transposons in clinical strains confirms the occurrence of “reverse transmission” from environmental to clinical settings (Grevskott et al., 2024).

Phage particles present in hospital wastewater can carry ARGs such as *bla*_*KPC*_ and *aadA*, with concentrations reaching 10^3^–10^4^ copies per milliliter. Although these phages do not autonomously express the ARGs they carry, they can inject these genes into new host cells during infection, thereby mediating gene transfer within the *Enterobacteriaceae* family (Pires et al., 2023; Santosaningsih et al., 2023).

Furthermore, sublethal concentrations of antibiotics, heavy metals (such as copper and zinc), and disinfectants (chlorine-containing compounds) can work together to significantly promote HGT. These factors can increase the transfer frequency of ARGs by 2–100 times (Hutinel et al., 2022; La Rosa et al., 2025). Genetic exchange between environmental ARB and clinical strains is becoming increasingly common. For instance, the tigecycline resistance gene cluster carried by an XDR *K. pneumoniae* ST16 strain in hospital wastewater differs from the sequence of clinical ST16 strains by only two single-nucleotide polymorphisms (SNPs). This close genetic similarity exacerbates the challenges associated with controlling clinical antibiotic resistance (Martins et al., 2022).

#### 6.2.3 Characteristics of ARGs in hospital wastewater and variations in treatment methods across different countries

The above-mentioned transmission routes are universal worldwide. However, due to differences in regional medication habits, processing technical levels, and regulatory systems, there are significant variations in the dominant types, abundances, and main treatment methods of ARGs in hospital wastewater across different countries. A detailed comparison is presented in Table 3.

**TABLE 3:** Antibiotic resistance genes (ARGs) in wastewater from different countries and mainstream treatment technologies.

Country/region	Dominant ARGs types	Mainstream wastewater treatment process	References
China	*bla_kp_c (Klebsiella pneumoniae), bla_oxa_, mcr-3.17 (Aeromonas veronii)*	Conventional activated sludge process + chlorination disinfection; MBR + UV disinfection adopted in some tertiary hospitals	Liu J. et al., 2025; Zhu et al., 2023
Brazil	*bla_kp_c, bla_ctx_–m_* (*Escherichia coli*, *Klebsiella pneumoniae*)	Centralized activated sludge process + sodium hypochlorite disinfection	Pires et al., 2023
South Africa	*tet*, *sul*, β-lactam ARGs (*Klebsiella pneumoniae*, *Escherichia coli*)	Simple primary treatment + chlorination disinfection (small institutions); activated sludge process (large hospitals)	Alam et al., 2022
Hungary	*bla_ctx_–m_, sul1* (*Escherichia coli*, *Enterococcus*)	Activated sludge process + anaerobic digestion	Mutuku et al., 2022
Japan	*bla_*kp*_c2* (*Citrobacter freundii*, *Klebsiella*)	Membrane Bioreactor (MBR) + ozone disinfection	Ota et al., 2022
United States	*ermB*, *qnrS*, *blaTEM* (*Staphylococcus aureus*, *Escherichia coli*)	Advanced oxidation (UV/H_2_O_2_) + activated sludge process	Silvester et al., 2025; Gönder et al., 2021
Germany (EU)	*sul1*, *tetM*, *intI1* (*Escherichia coli*, *Enterococcus*)	Biofilm process + sand filtration + ultraviolet disinfection	Lamba et al., 2017
India	*bla_*oxa*_–21*, *aacA3* (*Pseudomonas aeruginosa*, *Klebsiella pneumoniae*)	Conventional activated sludge process (no advanced treatment)	Anssour et al., 2016

### 6.3 Mutations and evolutions of ARGs

#### 6.3.1 Mutation mechanisms and enhanced antibiotic resistance

Point mutations in ARGs can significantly alter resistance levels: Zhu et al. (2023) reported that a 23-amino acid mutation in mcr-3.17, such as the loss of two β-sheets in the membrane-anchoring region, reduced the minimum inhibitory concentration (MIC) of colistin from 8 to 4 μg/mL. However, resistance could be restored through L-arabinose-induced expression. Additionally, the Val56Glu mutation in *bla_*CTX*–*M*–27_* increased the hydrolysis efficiency of ceftazidime by 1.8-fold, increasing the strain’s MIC from 4 to 16 μg/mL (Mutuku et al., 2022).

#### 6.3.2 Emergence of cross-resistance

Some mutations can lead to resistance to multiple classes of antibiotics. In *K. pneumoniae*, the A116S mutation in the *qnrB1* gene significantly increased the MICs of ciprofloxacin and levofloxacin. Specifically, the MICs increased from 0.25 and 0.5 μg/mL to 8 and 16 μg/mL, respectively, indicating cross-resistance to quinolones (Mutuku et al., 2022). Similarly, in *E. coli* ST38, the T35A mutation in *bla_*OXA*–244_* increased resistance to both imipenem and meropenem by twofold (Grevskott et al., 2024).

#### 6.3.3 Environmental spread and long-term risks of mutated ARGs

Mutated ARGs can rapidly spread via plasmids. For example, the *IncFIB* plasmid carrying *bla_*OXA*–244_* with the T35A mutation can be transferred to five different *Enterobacteriaceae* species during wastewater treatment, with a transfer frequency of 10^–6^ per recipient cell (Grevskott et al., 2024). Similarly, the *tmexD3.3* gene with the Val56Glu mutation in *K. pneumoniae* ST16 is integrated into the chromosome via Tn6855, enabling stable survival and spread in the environment (Martins et al., 2022).

Ecological and health risks: The spread of mutated ARGs can significantly alter the structures of environmental bacterial communities. Metagenomic analyses revealed that the proportion of antibiotic-resistant bacteria in aquatic environments adjacent to hospital wastewater discharge outlets reached 35%, which is in stark contrast to the mere 5% detected in control areas. This notable shift was accompanied by a 60% reduction in the abundance of susceptible bacterial strains (e.g., *Enterococcus faecalis*), which disrupted the natural ecological balance of the microbial community (Velazquez-Meza et al., 2025). In humans, ST11-type carbapenem-resistant *K. pneumoniae* (CRKP) originating from hospital wastewater could cause community-acquired infections with a treatment failure rate reaching 42%, which is significantly higher than that of clinical strains (25%) (Martins et al., 2022; Liu et al., 2023).

## 7 Long-term effects of antibiotic residues in hospital wastewater on ecosystems and biotic communities

Antibiotic residues in hospital wastewater, through continuous exposure, have multidimensional and often irreversible long-term effects on both the biophysical environment and various species. These impacts are reflected primarily in several key areas: imbalances in microbial community structure, disruptions in ecosystem functions, declines in biodiversity, and contamination of the food chain. These effects are interconnected and propagate through different levels, ultimately posing threats to the stability of ecosystems and human health.

### 7.1 Alterations in microbial community structure

Antibiotic residues in hospital wastewater significantly alter the compositions and functions of microbial communities through a dual mechanism: they inhibit susceptible bacteria while enriching resistant bacteria, thereby disrupting the original microbial balance of the ecosystem.

#### 7.1.1 Bidirectional selection of susceptible bacterial inhibition and enrichment of resistant bacteria

Antibiotics inhibit the growth of susceptible microorganisms through specific mechanisms. For example, ß-lactam antibiotics (such as penicillins and cephalosporins) interfere with bacterial cell wall synthesis, exerting strong inhibitory effects on both gram-positive and some gram-negative bacteria. Tetracyclines disrupt bacterial protein synthesis, thereby affecting the metabolic activities of susceptible bacteria. This inhibition leads to a significant decrease in the proportion of susceptible bacteria within the community. Moreover, resistant bacteria, whose resistance genes are acquired through mutation or HGT, proliferate extensively under antibiotic selective pressure (Zhang S. et al., 2025). For instance, in water bodies near hospital wastewater discharge areas, the proportion of MDR bacteria, such as *E. coli* and *Enterococcus* spp., is 3–5 times greater than that in control areas. These resistant bacteria can transfer resistance genes to other microorganisms through plasmid-mediated conjugation, thereby further expanding the spread of resistance (Mackul’ak et al., 2021).

#### 7.1.2 Systemic restructuring of community composition and function

Antibiotic residues not only alter microbial quantities but also lead to functional degradation of communities. Microorganisms with key ecological functions, such as organic matter decomposition and nitrogen fixation, are inhibited, causing disruptions in the functional chains of ecosystems (Cycoń et al., 2019). For example, in soil environments, when the concentration of oxytetracycline (OTC) reaches 200 mg/kg dry weight, nitrogenase activity is inhibited by 50% during the initial composting stage. In the later stages, the abundance of nitrogen fixation-related genes (*nifH*) decreases by 50%. Moreover, the richness and dominance of nitrogen-fixing bacterial communities decrease, having long-term effects on soil fertility maintenance (Sun et al., 2016). In aquatic environments, antibiotics can inhibit the activity and abundance of *Comammox Nitrospira*, altering the community structure of nitrifying bacteria. This process reduces nitrogen cycling efficiency and causes the accumulation of nitrates and nitrites, thereby exacerbating water quality deterioration (Liu et al., 2022).

### 7.2 Ecosystem functional dysregulation

Alterations in microbial community structure further trigger the dysregulation of ecosystem functions, which primarily manifests as decelerated organic matter decomposition, uncontrolled algal proliferation, and declining soil fertility, resulting in a vicious cycle of “functional dysregulation-environmental deterioration.”

#### 7.2.1 Significant reduction in organic matter decomposition efficiency

The activity of microorganisms responsible for organic matter decomposition, such as *Pseudomonas* spp. and Actinomycetes, is inhibited by antibiotics, thereby slowing the rate of material cycling in ecosystems. Studies have shown that when the concentration of tetracycline reaches 1,000 mg/kg, the abundance and diversity of active microorganisms in earthworm composting systems significantly decrease. This process results in a 30–40% reduction in the rate of organic matter maturation (Xia et al., 2019). In water bodies near hospital wastewater discharge areas, the rate of microbial organic matter decomposition is 25–30% lower than that in control areas, which leads to the accumulation of COD and exacerbation of eutrophication (Mora-Gamboa et al., 2022).

#### 7.2.2 Chain reactions triggered by algal overproliferation

The accumulation of organic matter provides a rich nutrient source for algae, while the weakened inhibitory effect of microorganisms on algae leads to massive algal proliferation (Nong et al., 2024). Excessive algal growth not only reduces water transparency and blocks light for submerged plants (for example, during *cyanobacterial* blooms in Lake Taihu, the coverage of submerged plants decreased by 60%) but also consumes a large amount of DO during decomposition after death. The DO concentration in the water can decrease below 0.5 mg/L within a short period, creating an anaerobic environment that threatens the survival of fish and other aquatic organisms. Moreover, the organic matter and nutrients released from algal decomposition further exacerbate eutrophication, resulting in a chain reaction of “algal proliferation—DO decline—organism death” (Liu et al., 2012).

#### 7.2.3 Persistent decline in soil fertility

Soil microorganisms are the core maintainers of soil fertility. Antibiotic residues, by inhibiting microbial activity, interfere with soil nutrient cycling. Low concentrations of tetracycline can inhibit the growth of soil bacteria and *Actinomyces*, reducing the rate of organic carbon mineralization and leading to insufficient accumulation of soil organic matter. Moreover, antibiotics inhibit the activity of nitrifying bacteria, reducing the conversion of ammonium nitrogen (NH^4+^) to nitrate (NO^3–^), decreasing the available nitrogen sources for plants, and affecting plant growth. Studies have shown that in soil treated with antibiotics, the number of nitrifying bacteria decreases by 40%, and the nitrogen transformation efficiency is reduced by 35% (Lucas et al., 2021).

### 7.3 Decline in biodiversity

Antibiotic residues can reduce biodiversity through direct toxicity and disruption of ecological functional chains, which manifests as the extinction of susceptible species, impaired growth and reproductive capabilities of organisms, and altered behavioral patterns.

#### 7.3.1 Regional decline in susceptible species

Long-term exposure to low concentrations of antibiotics can cause chronic poisoning in susceptible aquatic organisms, such as certain fish and invertebrates, leading to population decline and even extinction (Voitsitskiy et al., 2019). For example, at concentrations in the μg/L range, quinolone antibiotics (such as ofloxacin) can damage the immune system of carp larvae, reducing their disease resistance by 50% and inhibiting metabolic activity, which results in stunted growth. *Danio rerio* that are exposed long-term to 200 μg/L sulfamethoxazole show a 30% reduction in egg production, a 25% decrease in the larval survival rate, and an increased rate of developmental abnormalities in offspring, accelerating the decline of susceptible species (Sharma et al., 2021).

#### 7.3.2 Compression of biodiversity by the spread of resistant bacteria

Resistant bacteria present in hospital wastewater, such as MRSA and CRKP, can proliferate in the environment. These bacteria compete for resources and secrete antimicrobial substances, thereby squeezing the survival space of other organisms. Moreover, resistant bacteria can transfer resistance genes to opportunistic pathogens in the environment via HGT, increasing resistance across different species and further disrupting the balance of symbiosis and competition among organisms (Wachino, 2025). For instance, when the proportion of resistant bacteria in soil increases to 35%, the abundance of susceptible microorganisms (such as *Enterococcus faecalis*) decreases by 60%, leading to a simplification of soil biotic communities.

#### 7.3.3 Systemic impairment of organism growth and reproduction

The impacts of antibiotic residues on organism growth and reproduction are universally observed across species. In aquatic organisms, at concentrations ranging from the μg/L to mg/L scales, erythromycin can disrupt the primary defense and antioxidant systems of fish, causing cytotoxicity and neurotoxicity (Tong et al., 2025). This process results in a 40% reduction in *Danio rerio* weight gain and a 30% increase in reproductive cell damage rate (Jijie et al., 2021). In soil organisms, at concentrations of 50–500 mg/kg, sulfamethoxazole can reduce the cocoon production of earthworms (Dendrobaena veneta) by 50% and decrease the larval hatching rate by 45%. Earthworms respond to toxicity by reducing feeding and activity, which in turn affects soil aeration and organic matter decomposition (Žaltauskaitė et al., 2025).

#### 7.3.4 Abnormal changes in organism behavioral patterns

Antibiotic residues alter organismal behavioral patterns by interfering with the nervous and metabolic systems, reducing their survival ability. In water, low concentrations of tetracycline can slow fish swimming speed by 25%, increase their reaction time to food by 30%, and make their escape behavior sluggish when facing predators, thereby increasing predation risk (Amangelsin et al., 2023). In soil, tetracycline at 300 mg/kg can cause earthworms to exhibit significant avoidance behavior, reduce feeding by 40%, and inhibit the metabolic activity of nitrifying bacteria, leading to a 35% decrease in the soil nitrogen cycling rate (Lucas et al., 2021). This process creates a dual impact of “abnormal behavior-impaired function” (Simbanegavi et al., 2024; Timmerer et al., 2020).

In summary, the long-term impacts of antibiotic residues in hospital wastewater on the biophysical environment and species extend from the microbial level to macroecosystems and from single-function impairment to systemic imbalance. These impacts are characterized by their hidden nature, accumulation, and irreversibility. It is necessary to implement comprehensive measures, such as controlling antibiotic misuse at the source, upgrading wastewater treatment technologies, and establishing long-term monitoring systems, to curb the ongoing destruction of ecosystems.

### 7.4 Long-term effects on biological species

Antibiotic residues in hospital wastewater pose significant long-term and direct threats to biological species, including humans, through two major pathways: food chain contamination and health risks. In addition to altering microbial communities, disrupting ecological functions, and reducing biodiversity, these impacts are characterized by their “transferability and accumulation,” ultimately forming a chain reaction of “environmental exposure-bioaccumulation-health impairment.”

#### 7.4.1 Food chain contamination: bioamplification and transfer of antibiotics

Antibiotic residues can enter organisms through aquatic and soil environments and are transferred up the food chain via bioamplification, ultimately threatening top predators, such as humans.

Pollution Source: The concentration of antibiotic residues in hospital wastewater is significantly greater than that in natural water bodies. For instance, the concentration of sulfonamide antibiotics can reach 20.6 μg/L. Even after conventional wastewater treatment processes, antibiotics such as ciprofloxacin and sulfamethoxazole cannot be completely removed. These residual antibiotics become the core source of food chain contamination (Chu et al., 2024).

Transfer Process: Antibiotics spread through the pathway of “environment-primary producers-primary consumers-secondary consumers-top consumers.” Zooplankton first absorb antibiotics from the water and are then consumed by small fish. The antibiotics in small fish are transferred up the food chain to larger fish and eventually enter the human body through the consumption of contaminated aquatic products. During this process, antibiotics accumulate through bioamplification. For example, the concentration of antibiotics in large fish can be 10–100 times greater than that in water, significantly increasing the risk of human intake.

Direct Damage to Organisms: Antibiotic residues can directly damage organisms at each link of the food chain. After consuming zooplankton containing antibiotics, aquatic organisms (such as fish) may experience growth inhibition and immune system impairment. When these damaged organisms enter higher trophic levels, they not only transfer antibiotics but also may affect the stability and integrity of the food chain because of their abnormal physiological functions (Morillas-España et al., 2025).

#### 7.4.2 Health risks: microbial imbalance and threat of antibiotic resistance

Long-term exposure to low concentrations of antibiotics or intake through the food chain poses multidimensional health risks to organisms, especially humans, primarily in the form of imbalances in gut microbiota and increased risks of resistant bacterial infections.

Impact on the Gut Microbiota: Gut microbiota are crucial for maintaining normal physiological functions in humans, especially for the initiation and maturation of the adaptive immune system (Zhou et al., 2021). These microbiota achieve interspecies cooperation through a metabolite-dependent network (de Vos et al., 2022). Long-term exposure to antibiotics can disrupt this balance in several ways: (1) reducing gut species diversity, such as decreasing the abundance of susceptible bacteria (e.g., *Bifidobacterium* and *Lactobacillus*); (2) altering microbial metabolic activity, affecting the synthesis of beneficial metabolites such as short-chain fatty acids; and (3) selectively enriching resistant microorganisms. This process can ultimately lead to antibiotic-associated diarrhea and recurrent *Clostridium difficile* infections. The risks are more pronounced in early childhood, potentially inducing gastrointestinal diseases, immune dysfunction, and neurocognitive developmental issues with irreversible effects (Ramirez et al., 2020).

Risk of Resistant Bacterial Infections: Long-term exposure to low concentrations of antibiotics increases the likelihood of organisms being infected with resistant bacteria. Resistant bacteria in the environment (such as MRSA and CRKP) can enter the human gut through diet and drinking water and can exchange genes with the gut microbiota. Moreover, the suppression of susceptible gut bacteria by antibiotics provides a niche for resistant bacteria to colonize and proliferate. If human immunity is compromised, these resistant bacteria may cause infections. Owing to their resistance to multiple antibiotics, these bacteria can significantly reduce the effectiveness of clinical treatments, increasing the risk of severe infections and mortality. Additionally, antibiotics may harm human reproductive and developmental systems through endocrine disruption and genotoxic mechanisms, further expanding the scope of health risks.

In summary, the long-term effects of antibiotic residues in hospital wastewater on biological species are transferred from the environment to organisms through food chain contamination and are then transformed from organism physiology to disease through health risks. These impacts not only threaten the survival and health of individual species but also may trigger cross-species health crises through food chain associations. This finding highlights the urgency of controlling antibiotic emissions from hospital wastewater at the source and strengthening end-of-pipe treatment.

## 8 Antibiotic and antibiotic-resistant-bacteria detection and monitoring

### 8.1 Advances and limitations in antibiotic analytical technologies

Hospital wastewater is among the primary sources of antibiotic residues in the environment. Although antibiotics are typically present in environmental waters at trace concentrations (ng/L), their continuous discharge and bioaccumulative properties have led to global environmental issues. These residues have been detected in surface water, groundwater, and even drinking water. For instance, in inland European surface waters, the concentration range of sulfamethoxazole is 0.5–17 μg/L, and that of clarithromycin is 0.5–16 μg/L. Downstream of hospital wastewater discharge outlets in some Chinese cities, fluoroquinolone antibiotics (such as ciprofloxacin) can reach concentrations of 2–5 μg/L (Baralla et al., 2021; Nappier et al., 2020).

Hospital wastewater is characterized by a diverse range of antibiotics and significant concentration fluctuations. Inpatients often use large amounts of antibiotics for therapeutic needs, and 30%–90% of these drugs are excreted in urine and feces in their original form or as metabolites, where they enter the wastewater treatment system (Tao et al., 2025). However, existing treatment facilities have limited effectiveness in terms of removing most antibiotics. Conventional activated sludge processes achieve only a 20–40% removal rate for β-lactam antibiotics and less than 30% for macrolides (such as erythromycin). Even with ozonation at 0.15 mg/L, resistant genes (such as *oqxB* and *qepA*) cannot be completely removed and may even increase in relative abundance because of selection pressure (Abbott et al., 2020).

Moreover, there are distinct differences between hospital- and agricultural/livestock-sourced antibiotic residues. Hospital sources are primarily cephalosporins and fluoroquinolones, often cooccurring with disinfectants (such as chlorine) to form complex pollution. In contrast, agricultural sources are mainly tetracyclines and penicillins. This difference makes the antibiotic residues near hospital wastewater discharge outlets pose a greater ecological risk. For example, there are significantly higher co-occurrence rates of resistance genes and pathogenic bacteria (Abbott et al., 2020; Siedlecka et al., 2021).

To date, the detection technology for residual antibiotics in hospital wastewater has significantly evolved. It has transitioned from high-performance liquid chromatography (HPLC) to liquid chromatography-tandem mass spectrometry (LC-MS/MS), achieving a detection limit as low as 0.1 ng/L. This advancement enables the simultaneous quantification of dozens of antibiotics (Angeles and Aga, 2018; Fabregat-Safont et al., 2023). For example, the combination of solid-phase extraction (SPE) and LC-MS/MS can detect 32 types of antibiotics, including sulfonamides, quinolones, and macrolides, from a 100-mL sample of hospital wastewater. Moreover, the application of metagenomic sequencing technology, in conjunction with the comprehensive antibiotic resistance database (CARD), has opened new avenues. Scholars can identify antibiotic resistance genes (e.g., the *bla*_*OXA*_ gene related to β-lactam resistance) and reveal their diversity and association with microbial communities (Miłobedzka et al., 2022).

Despite continuous advancements in detection technologies, two major limitations remain. First, the lack of standardized detection methods: the significant differences in sample pretreatment methods (such as types of extraction columns and elution solvents) and instrumental parameters used in different studies, result in poor data comparability. For instance, the concentration of ciprofloxacin in the same hospital wastewater sample may vary by 2–3 times when C18 and HLB extraction columns are used (Opriş et al., 2013). Second, the scope of detection is limited: existing research is focused mostly on common antibiotics, but coverage of new types of antibiotics such as carbapenems and compound formulations is insufficient. This research gap may lead to an underestimation of the actual residual risk (Angeles and Aga, 2018; Li et al., 2023).

### 8.2 Shortcomings and optimization directions of antibiotic monitoring systems

The limitations of current monitoring systems significantly hinder the control of antibiotic pollution. To date, most countries have yet to include antibiotics in hospital wastewater as part of their routine environmental monitoring indicators. Only the European Union and the Yangtze River Delta region in China have established quarterly monitoring systems (Mackul’ak et al., 2021). Moreover, there are substantial flaws in the data sharing mechanisms for antibiotic resistance (AR) monitoring. In most countries, data are circulated only internally or among cooperating institutions, lacking cross-regional and standardized sharing platforms. This fragmentation makes it difficult to integrate and analyze data from different studies or regions (Gholipour et al., 2024). For example, a resistance monitoring study of urban wastewater in 10 European countries revealed that the average detection rate of resistant *E. coli* was 12% in northern European countries but 35% in southern European countries. However, owing to non-uniform sampling methods, detection standards, and data formats, it is impossible to clearly determine whether these differences stem from regional medication habits, environmental transmission, or the monitoring methods themselves. Consequently, tracing the cross-border transmission pathways of resistance genes remains a significant challenge (Gholipour et al., 2024).

Future improvements to detection and monitoring systems should focus on three key areas. First, promoting the standardization of detection methods by unifying sample pretreatment procedures and quality control standards is essential. Second, establishing a comprehensive monitoring network that covers the entire chain from “hospitals—municipal pipelines—receiving water bodies” will enhance the ability to track and manage antibiotic pollution. Third, combining biological monitoring using indicator organisms such as *Danio rerio* and algae with predictive modeling tools such as the soil and water assessment tool (SWAT) model can provide a more comprehensive assessment of residual risks and a scientific basis for control measures.

Antibiotic residues in hospital wastewater pose a serious threat to ecosystems because they affect the physiological functions and community structures of aquatic organisms. The current situation, which is characterized by high residual levels and difficulty in treatment removal, is exacerbated by the limitations of detection and monitoring systems. This limitation further increases the difficulty of effectively controlling antibiotic pollution. Therefore, reducing the misuse of antibiotics at the source, upgrading wastewater treatment technologies, and improving detection and monitoring systems are imperative. By addressing these issues from multiple dimensions, we can reduce the environmental risks associated with antibiotic residues and protect aquatic ecosystems.

### 8.3 Antibiotic resistance issues in wastewater treatment processes

#### 8.3.1 Limitations of existing treatment technologies

Chlorine-containing disinfectants, such as sodium hypochlorite (NaClO), which are commonly used in hospital wastewater treatment, have limited effects on antibiotic-resistant bacteria. For example, the minimum inhibitory concentration (MIC) of NaClO for CRKP is ≥ 400 μg/mL, with 72.04% of strains surviving at this concentration (Liu et al., 2023). Compared with susceptible strains, XDR *K. pneumoniae* ST16 is 3–5 times more resistant to chlorine disinfectants. Moreover, sublethal exposure to chlorine can increase the biofilm formation ability (the OD value can increase by 2.1 times), further reducing the efficacy of disinfection (Martins et al., 2022).

Wastewater treatment processes are often unable to completely remove ARGs and may even lead to their enrichment. A study by Mutuku et al. (2022) on hospital wastewater in Hungary revealed that the abundance of *bla_*CTX*–*M*_* in activated sludge was 2.3 times that in the influent, and the MAR index in digested sludge reached 0.56 (0.50 for the influent). Traditional chlorination removed only 30–40% of free bla_*KPC*_, and undegraded gene fragments could enter environmental bacteria via transduction (Hutinel et al., 2022).

#### 8.3.2 Directions for technological improvement and process optimization

Advanced oxidation processes, such as UV/H_2_O_2_, can effectively destroy the cell membranes and DNA of resistant bacteria through the action of hydroxyl radicals. This technology achieves a 99.9% removal rate for CRKP and reduces the abundance of ARGs by 1–2 orders of magnitude (Grevskott et al., 2024; Velazquez-Meza et al., 2025). To further increase treatment efficacy, the integration of digital PCR for real-time monitoring of ARG residuals is recommended. For instance, the detection limit for *bla*_*NDM*_ can be as low as 10 copies/mL, ensuring the safety of the treated effluent (Erler et al., 2024).

The combined use of “adsorption-biodegradation” processes is highly recommended. Initially, activated carbon is employed to adsorb antibiotics, achieving a removal rate of ≥ 90%. Subsequently, residual drugs are degraded by functional bacterial communities, such as *Pseudomonas* spp. This approach not only reduces the selective pressure on resistant bacteria but also mitigates the risk of ARG enrichment, thereby optimizing the overall treatment process (Zahra et al., 2023).

## 9 Conclusion

We systematically highlight that hospital wastewater treatment systems are confronted with four major core deficiencies. First, there is a lack of essential facilities and outdated designs. A significant number of primary treatment units, such as screens and equalization tanks, are missing. Moreover, the static nature of these designs fails to keep pace with the exponential growth in medical demand. Second, equipment aging and inadequate O&M are prevalent issues. More than 60% of small medical institutions experience at least two equipment failures per year. The lack of preventive maintenance leads to frequent disruptions in the treatment process. Third, there are dual constraints of funding and space. Small institutions face high per-ton investment costs and low net profits. Additionally, there is no available land for expansion, limiting their ability to upgrade or expand their treatment facilities. Fourth, there is a systemic deficit in human capital. More than 50% of institutions lack dedicated certified operators, and a staff turnover rate of 30% exacerbates technical deficiencies.

These structural flaws result in long-term overloading and inefficient operation of treatment systems. Consequently, high concentrations of antibiotics (such as β-lactams and fluoroquinolones) and ARGs (such as *bla*_*KPC*_ and *mcr*) are discharged into the environment. Pollutants spread through multiple media, including water, soil, food chains, and aerosols, creating a bidirectional resistance transmission loop between the environment and clinical settings. At the ecological level, residual antibiotics reshape microbial communities through selective pressure. Antibiotics inhibit susceptible bacteria (such as nitrifying and nitrogen-fixing bacteria), enrich resistant bacteria, and interfere with the physiological metabolism and reproduction of organisms across multiple trophic levels, such as algae and fish. This process leads to a 40% decrease in ecosystem material cycling efficiency and a 35% decrease in biodiversity. At the health level, ARGs accelerate mutations through HGT, resulting in a 42% treatment failure rate for resistant bacterial infections in community-acquired cases. This phenomenon poses a severe challenge to the existing antimicrobial drug system. Moreover, the current monitoring and governance systems are hindered by a lack of standardized methods, insufficient data sharing, and incomplete full-chain coverage. These shortcomings further restrict the effectiveness of pollution control efforts.

## 10 Future outlook

To effectively address the core challenges of hospital wastewater pollution, a paradigm shift from “end-of-pipe reduction” to “life cycle risk prevention and control” is essential. This transition requires breakthroughs in five key areas.

### 10.1 Technology upgrades and process innovation

First, “modular-elastic” designs should be promoted, reserving ≥ 30% capacity beyond baseline demand to accommodate dynamic growth in hospital beds and outpatient volumes. Second, advanced treatment processes such as UV/H_2_O_2_ advanced oxidation, activated carbon adsorption–biodegradation, and membrane separation-disinfection coupling should be integrated. These technologies can achieve antibiotic removal rates > 90% and reduce the abundance of ARGs by 1–2 orders of magnitude. Third, decentralized governance systems using MBR and MABR (membrane aerated biofilm reactor) “source-sink balance” systems should be implemented. This approach reduces the reliance on centralized pumping stations and enhances the resilience of small medical institutions.

### 10.2 Monitoring, early warning, and digital governance

The “full-chain multidimensional” monitoring system should be enhanced. First, a standardized monitoring network covering hospitals, municipal pipelines, and receiving water bodies should be established. Sample pretreatment methods (e.g., extraction column types and elution solvents) and quality control processes for LC-MS/MS detection should be unified to improve data comparability. Second, multiple monitoring technologies, including metagenomic sequencing (to analyze ARG diversity), digital PCR (for precise quantification of mutant genes), and *Danio rerio*/algae bioindicators (to assess ecological toxicity), should be combined. This integration enables real-time tracking of resistance gene mutation hotspots. Third, AI-driven predictive models, such as SWAT–ARG coupling models, should be developed to provide 72-h advance warnings of pollution events and support emergency response efforts.

### 10.3 Policy-economic-social synergistic mechanisms

A “rigid constraints-resource assurance-capacity enhancement” safeguard system is constructed. First, policy enforcement should be strengthened by incorporating essential units such as screens and equalization tanks into mandatory standards for medical facility construction. Moreover, non-negotiable safety baselines should be set to ensure compliance. Second, regionally intensive maintenance centers should be established to centrally allocate O&M resources. A microcertification quarterly training system (2 h of online courses + practical assessment per quarter) is implemented to address human capital deficits. Third, economic incentives should be innovated by leveraging green finance and carbon trading tools to internalize the external health costs of resistance. Guide social capital investment in treatment technology upgrades.

### 10.4 Research and intervention on ARG mutations

Basic and applied research on mechanism dissection and targeted prevention and control should be advanced. First, a globally shared ARG mutation database focusing on the ecological adaptability mechanisms of key mutation sites in genes such as mcr and *bla*_*OXA*_ (e.g., Val56Glu and T35A) should be established. Second, targeted intervention technologies, such as CRISPR-Cas9-based ARG knockout techniques, should be developed to block the horizontal transfer of high-risk genes. Third, collaboration among medical, environmental, and agricultural departments should be strengthened to curb the cyclical spread of resistance among humans, animals, and environments.

### 10.5 Public participation and international cooperation

**FIGURE 7:**
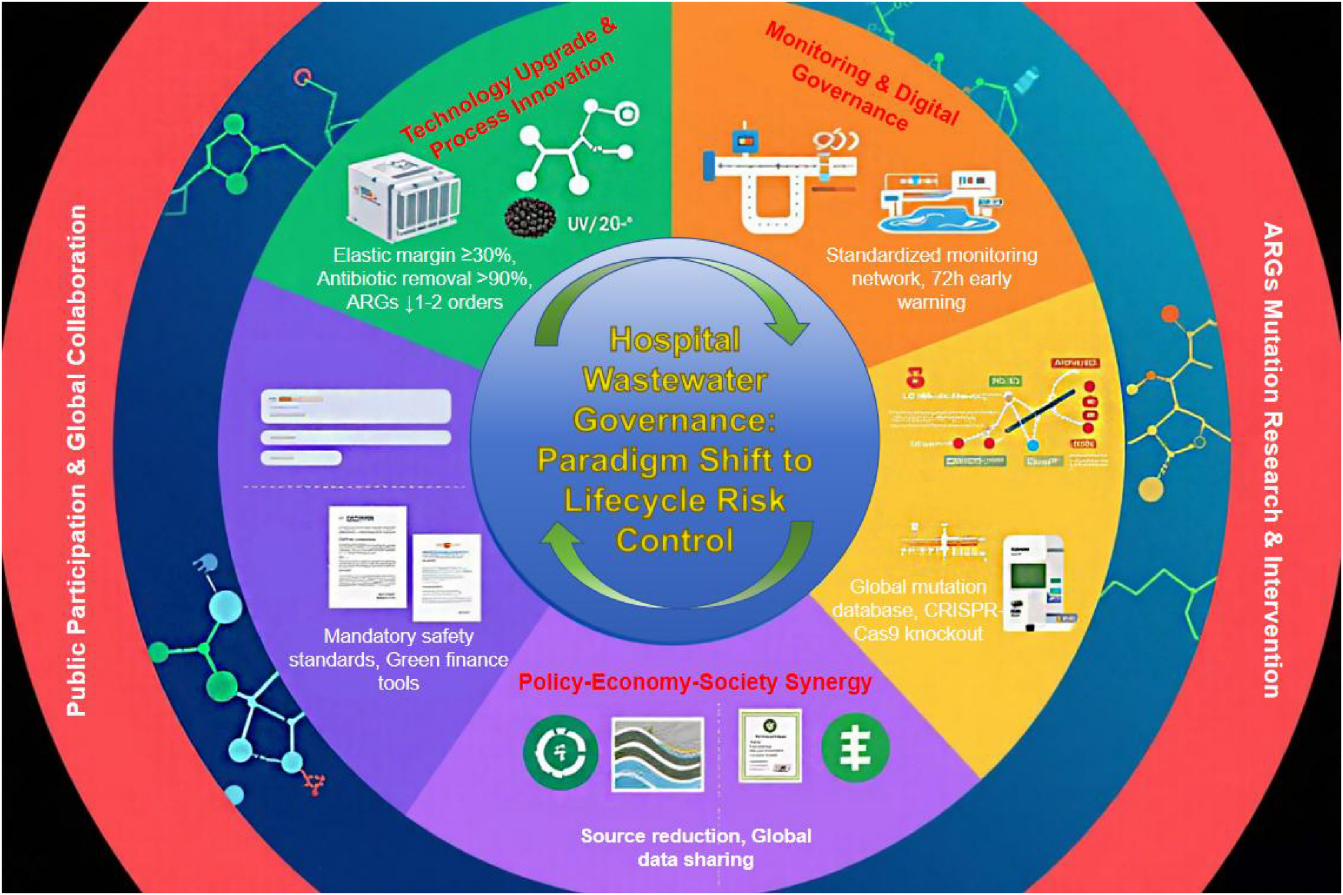
Hospital wastewater governance: paradigm shift to lifecycle risk control.

The governance dimension of “source reduction-global collaboration” should be expanded. As Figure 7 (the paradigm shift to hospital wastewater lifecycle risk control) illustrates, this dimension encompasses multiple synergistic measures. First, source control should be strengthened via healthcare worker training to standardize antibiotic prescriptions and patient education to reduce self-medication, cutting antibiotic emission loads at the source. Second, the global antimicrobial resistance surveillance system (GLASS-WGS) should be used to share ARG monitoring data and codevelop transnational emission standards. Third, capacity building should be supported through south-south cooperation and technology transfer, providing modular treatment equipment and O&M training to low- and middle-income countries to increase global hospital wastewater governance levels. Together with other key measures (as outlined in Figure 6), these actions enable a comprehensive, sustainable approach to hospital wastewater pollution management, safeguarding both environmental and public health.

Hospital wastewater governance: Paradigm shift to lifecycle risk control. This figure presents a comprehensive framework for hospital wastewater governance, structured around four interconnected pillars: technology upgrades and process innovation (≥ 30% elastic margin, > 90% antibiotic removal), monitoring and digital governance (standardized networks, 72-h early warning), policy-economy-society synergy (mandatory standards, green finance), and public participation and global collaboration. This integrated approach enables sustainable, lifecycle-based risk management.

In conclusion, hospital wastewater pollution has transcended the scale of a local technical issue to become a global crisis that intertwines ecology and health. To break the vicious cycle of pollution resistance, it is imperative to pursue technological innovation to overcome governance bottlenecks, undertake institutional restructuring to bolster safeguard capabilities, and foster multiparty collaboration to solidify governance efforts. Only through these concerted actions can we achieve multiple goals of safe hospital wastewater discharge, healthy and stable ecosystems, and sustainable public health.
